# In Vitro and In Vivo Evaluation of Nitroxoline as an Effective Antimicrobial Alternative to Poultry Production

**DOI:** 10.3390/antibiotics15010062

**Published:** 2026-01-06

**Authors:** Yuqing Zhou, Maria M. Trush, Lewis Ibbotson, Laura Espina, Aditya Kumar Lankapalli, Alistair J. M. Farley, Huangwei Song, Congming Wu, Xingyuan Cao, Xi Xia, Charlotte J. Gray-Hammerton, Alice Moorey, Amelie Delaitre, George Siegwart, Shaolin Wang, Yang Wang, Jianzhong Shen, Christopher J. Schofield, Timothy R. Walsh

**Affiliations:** 1 Department of Biology and the Ineos Oxford Institute for Antimicrobial Research, University of Oxford, South Parks Road, Oxford OX1 3RE, UK; mariia.trush@biology.ox.ac.uk (M.M.T.); aditya.lankapalli@biology.ox.ac.uk (A.K.L.); charlotte.gray-hammerton@biology.ox.ac.uk (C.J.G.-H.); alice.moorey@biology.ox.ac.uk (A.M.); amelie.delaitre@biology.ox.ac.uk (A.D.); george.siegwart@biology.ox.ac.uk (G.S.); 2 Department of Chemistry and the Ineos Oxford Institute for Antimicrobial Research, University of Oxford,12 Mansfield Road, Oxford OX1 3TA, UK; lewis.ibbotson@chem.ox.ac.uk (L.I.); alistair.farley@chem.ox.ac.uk (A.J.M.F.); christopher.schofield@chem.ox.ac.uk (C.J.S.); 3 Fundación Agencia Aragonesa para la Investigación y el Desarrollo (ARAID), Avenida Ranillas 1D, 50018 Zaragoza, Spain; espina@unizar.es; 4 College of Veterinary Medicine, China Agricultural University, No. 2 Yuanmingyuan West Road, Beijing 100193, China; hwei_song@126.com (H.S.); wucm@cau.edu.cn (C.W.); cxy@cau.edu.cn (X.C.); xxia@cau.edu.cn (X.X.); shaolinwang@outlook.com (S.W.); wangyang@cau.edu.cn (Y.W.); sjz@cau.edu.cn (J.S.)

**Keywords:** nitroxoline, antimicrobial alternative, poultry production, in vitro antibacterial activity, in vivo efficacy, pharmacokinetics, withdrawal time, gastrointestinal microbiota

## Abstract

Background: Antimicrobial resistance is a major global challenge that is exacerbated by extensive antibiotic use in livestock farming. Identifying effective alternatives to widely used human antibiotics in animal production is vital to safeguard vital human medicines and ensure sustainable food systems. Here we describe studies identifying nitroxoline (NTX) as a promising antimicrobial candidate for use in poultry production. Methods: The antibacterial activity and resistance potential of NTX were assessed in vitro. In vivo studies in chickens evaluated tolerance, therapeutic efficacy in *Salmonella*-infected birds, pharmacokinetics, tissue residue depletion, growth performance, and effects on caecal microbiota. NTX was administered in-feed at different dose levels. Pharmacokinetic parameters and withdrawal periods were determined, and caecal microbiota composition was analysed using ribosomal RNA 16S sequencing. Results: NTX exhibits potent broad-spectrum antibacterial activity in vitro and low levels of resistance. NTX is well-tolerated in chickens at 500 mg/kg in-feed for 7 days and substantially reduces liver bacterial loads at 100 mg/kg in *Salmonella*-infected chickens. Pharmacokinetic and residue analyses reveal NTX manifests rapid absorption and distribution, high oral bioavailability (86%), and efficient tissue clearance with a 17-day withdrawal period required for skin-plus-fat clearance. NTX supplementation is associated with increased weight gain and improved feed efficiency compared to the control group, with performance comparable to chlortetracycline. Microbiota analysis indicates modulation of caecal bacterial communities, including increased *Faecalibacterium* and *Lactobacillus*. Conclusions: These results indicate that NTX is a viable alternative to important human antibiotics widely deployed in poultry production, offering a potential approach to minimise antimicrobial resistance whilst maintaining animal health and food biosafety.

## 1. Introduction

Following the widespread introduction of effective antibiotics for human use in the 20th century, the same compounds were used in the meat industry to reduce disease and increase yields [1,2]. Multiple antibiotics are now used in livestock farming for disease prevention (prophylaxis), disease control (metaphylaxis), and therapeutic purposes (to reduce diseases and improve animal welfare) [3,4]. In some regions, antibiotics are routinely administered for non-medicinal purposes, in particular growth promotion and feed efficiency enhancement [4,5,6]. In poultry farming, antibiotics are frequently deployed to treat intestinal infections, including colibacillosis and necrotic enteritis, which are often caused by *Salmonella* spp., *Escherichia coli*, or *Clostridium perfringens* [7,8].

Global antimicrobial use (AMU) in food-producing animals is projected to increase by 67% from 2010 to 2030 [9,10]. This increase is largely driven by an escalating global demand for animal protein from an expanding population, leading to intensive livestock production that heavily relies on antibiotics, with the growth in poultry production and associated antibiotic use being a particular issue [11]. Many antibiotics used in agriculture, including tetracyclines, macrolides, beta-lactams, and fluoroquinolones, are vital in human medicine, raising concerns about the transfer of resistant bacteria and resistance genes between animals and humans [12,13].

During the 2024 United Nations General Assembly High-Level Meeting (UNGA HLM) on antimicrobial resistance (AMR), member states agreed to a declaration committing countries to a clear set of targets and actions to globally tackle AMR [14]. One of these was to meaningfully reduce the quantity of antimicrobials used in agri-food systems by 2030 [15]. This, in part, is to be achieved by better preventive strategies, including animal vaccination, good husbandry practices, biosecurity, and water, sanitation and hygiene (WASH); however, these strategies are proving difficult to achieve in much of the agriculture sector [16]. Repurposing of approved antibiotics that are not widely used in clinical medicine has emerged as a promising strategy to reduce AMR caused by the use of human antibiotics in farming [17,18].

With the aim of developing antibiotics for use in poultry production, we carried out screening of potential antibiotics using an assay involving twenty-five animal-specific pathogens. The screening results identified 5-nitroquinolin-8-ol (nitroxoline, NTX) as a promising candidate drug for use in poultry production.

Interest in NTX has recently grown due to its versatile medicinal properties, including antiangiogenic, anticancer, antibacterial, antifungal, antiviral, and antiparasitic activities, coupled with a clinically acceptable safety profile [19,20,21,22,23]. Kudera et al. have reported on the efficacy of NTX against seventeen diarrheal bacterial species, including *Bacillus cereus*, *Clostridial* species, *E. coli*, and *Shigella flexneri* (minimum inhibitory concentrations (MICs): 2–4 µg/mL) [24]. NTX also showed good efficacy against various multidrug-resistant (MDR) organisms such as carbapenemase-producing Enterobacterales, methicillin-resistant *Staphylococcus aureus* and drug-resistant *Neisseria gonorrhoeae* [25,26,27,28]. Despite its longstanding, though limited clinical use, NTX is reported to show minimal resistance development in *E. coli*, the most frequently encountered bacterial uropathogen [23,28].

NTX is a hydroxyquinoline derivative whose core antimicrobial activity has been linked to its metal-chelating properties [19]. By interacting with divalent metal ions such as Fe^2+^, Zn^2+^, Cu^2+^, and Mn^2+^, NTX disrupts metal-dependent bacterial enzymes and essential metabolic processes [25,26,29]. Recent studies have shown that NTX disrupts biofilm formation, inhibits metallo-β-lactamases, and perturbs redox and energy metabolism, consistent with a multi-target mode of action that may contribute to its broad-spectrum activity and low propensity for development of stable resistance [19,25,26,28].

In 2017, NTX was approved as a treatment option for uncomplicated urinary tract infections (UTIs) in Germany, alongside fosfomycin, nitrofurantoin, pivmecillinam, and trimethoprim [30]. The European Committee on Antimicrobial Susceptibility Testing (EUCAST) established NTX breakpoints for *E. coli* in uncomplicated UTIs at 16 mg/L [31]. Notably, NTX resistance development occurred more slowly than with the other tested antibiotics, including pivmecillinam, ciprofloxacin, and trimethoprim [32]. Recent studies report that the MDR efflux system *emrRAB* contributes to NTX resistance in *E. coli* and *Klebsiella pneumoniae*, with resistance mutations affecting bacterial fitness [32,33].

Despite limited evidence on the use of NTX to treat secondary bacterial infections associated with colibacillosis and salmonellosis in poultry in Korea [34], details on its efficacy and safety in animals are lacking. Here we describe studies on NTX that reveal its in vitro potency, in vivo tolerability, in vivo oral pharmacokinetics, tissue drug withdrawal time, efficacy in *Salmonella*-infected chickens, effects on chicken growth performance and impact on normal chicken intestinal microbiota.

## 2. Results

### 2.1. Identification of NTX and Its In Vitro Antibacterial Activity

A compound library was assembled following a comprehensive search of available antibiotic compound libraries and optimised through a comprehensive literature review. Following a preliminary screen, structurally related analogues of promising hits were purchased or synthesised in-house (Table S1, Supplementary Materials). The antibacterial activity of these compounds was initially tested against fifteen different species known for causing disease states in food-producing animals, comprising a panel of twenty-five reference strains, including a total of sixteen Gram-negative and nine Gram-positive bacteria under their optimum growth conditions (Table S2, Supplementary Materials). The MICs of each compound were determined using the Clinical and Laboratory Standards Institute broth protocol (CLSI) [35]. For the initial screen, the antimicrobial activity of compounds was considered of interest if their MICs were 16 µg/mL or lower.

**Figure 1:**
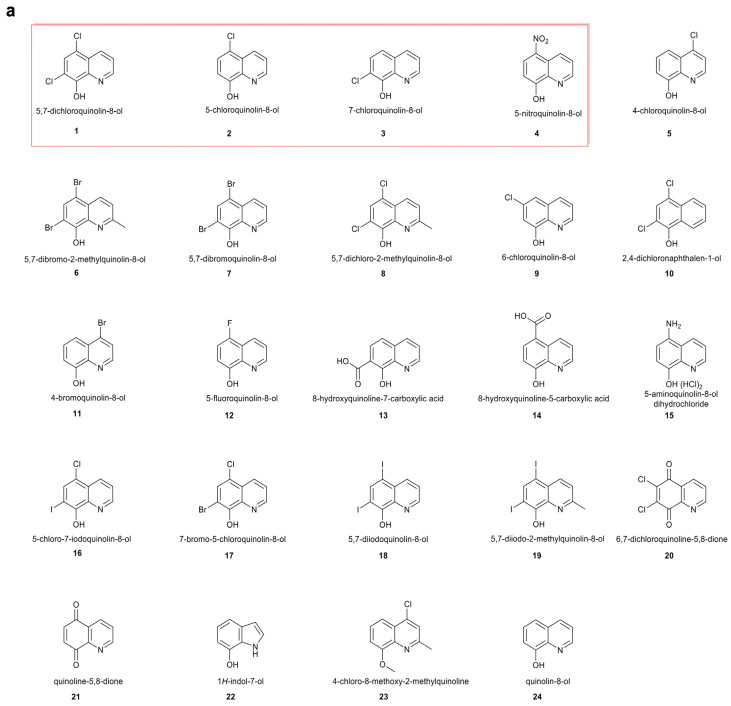

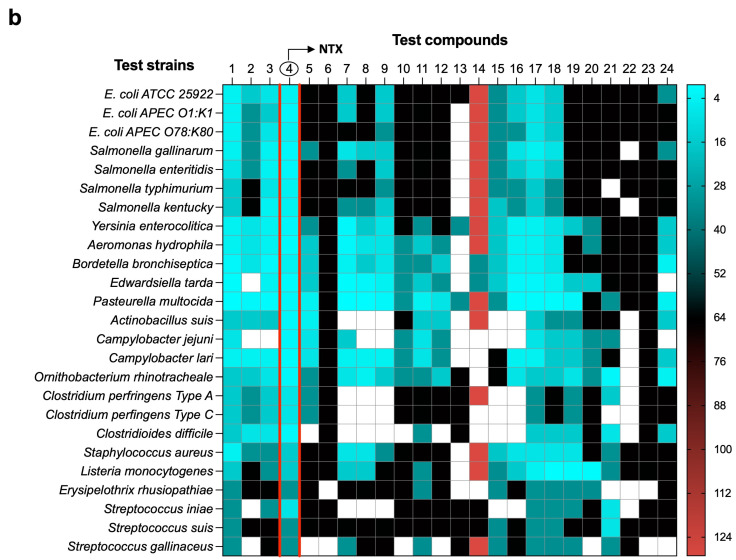
Microbiological screening of selected compounds versus animal pathogens. (**a**) Structures of screened compounds. (**1**) 5,7-dichloroquinolin-8-ol, (**2**) 5-chloroquinolin-8-ol, (**3**) 7-chloroquinolin-8-ol, (**4**) 5-nitroquinolin-8-ol (NTX), (**5**) 4-chloroquinolin-8-ol, (**6**) 5,7-dibromo-2-methylquinolin-8-ol, (**7**) 5,7-dibromoquinolin-8-ol, (**8**) 5,7-dichloro-2-methylquinolin-8-ol, (**9**) 6-chloroquinolin-8-ol, (**10**) 2,4-dichloronaphthalen-1-ol, (**11**) 4-bromoquinolin-8-ol, (**12**) 5-fluoroquinolin-8-ol, (**13**) 8-hydroxyquinoline-7-carboxylic acid, (**14**) 8-hydroxyquinoline-5-carboxylic acid, (**15**) 5-aminoquinolin-8-ol dihydrochloride, (**16**) 5-chloro-7-iodoquinolin-8-ol, (**17**) 7-bromo-5-chloroquinolin-8-ol, (**18**) 5,7-diiodoquinolin-8-ol, (**19**) 5,7-diiodo-2-methylquinolin-8-ol, (**20**) 6,7-dichloroquinoline-5,8-dione, (**21**) quinoline-5,8-dione, (**22**) 1*H*-indol-7-ol, (**23**) 4-chloro-8-methoxy-2-methylquinoline, (**24**) quinolin-8-ol. Compounds were from commercial suppliers (Sigma-Aldrich, Inc., St. Louis, MO, USA; Fluorochem Ltd., Derbyshire, United Kingdom; Tokyo Chemical Industries, Tokyo, Japan; MedChem express, Monmouth Junction, NJ, USA) and were used without further purification. Three structurally related hit compounds (5,7-dichloroquinolin-8-ol, 5-chloroquinolin-8-ol, and 7-chloroquinolin-8-ol) and the lead candidate 5-nitroquinolin-8-ol (NTX) are highlighted within a red rectangular box to indicate compounds selected for further evaluation based on shared structural features. (**b**) Heatmap depicting the antibacterial activities of the selected compounds against test strains under optimised conditions defined in Table S2 (Supplementary Materials). Compound **4**, nitroxoline (NTX), is highlighted by two red lines in (**b**). The colour intensity represents the minimum inhibitory concentration (MIC, µg/mL) as indicated by the key. Empty cells denote missing data. The heatmap was generated using GraphPad Prism 10.0.3.

Some of the hit compounds (5,7-dichloroquinolin-8-ol, 5-chloroquinolin-8-ol, and 7-chloroquinolin-8-ol) (compound **1**–**3**, Figure 1a) are sold commercially as halquinol, a long-established veterinary treatment that is widely used in pig and poultry production in many countries [35,36], making them attractive for further investigation. Building on the shared hydroxyquinoline core of these compounds, we conducted structure-activity relationship (SAR) studies with a series of hydroxyquinoline derivatives to identify promising candidates for further evaluation (Figure 1a). Among the tested analogues, NTX (compound **4**, Figure 1a) emerged as the most promising candidate; it was found to have potent activity against both Gram-negative and Gram-positive food-producing animal pathogens, with MICs ranging from 1 to 4 µg/mL and 2–32 µg/mL, respectively (Figure 1b). 5-Chloro-7-iodoquinolin-8-ol (compound **16**), 7-bromo-5-chloroquinolin-8-ol (compound **17**), and 5,7-diiodoquinolin-8-ol (compound **18**) exhibited activity comparable to the original hit-compound, 5,7-dichloroquinolin-8-ol (compound **1**) (Figure 1b), differing only in substitutions on the aromatic ring (Figure 1a), but were not pursued further because of the presence of iodine or bromine atoms in them, coupled with the extensive safety data available for NTX (compound **4**).

Following the identification of NTX as a promising candidate from the initial screen, its activity was evaluated against an extended panel of poultry-sourced *E. coli* (*n* = 66) and *Salmonella enterica* (*S. enterica*) strains (*n* = 22) with diverse resistance phenotypes. NTX effectively inhibited the growth of all the tested isolates, with MICs ranging from 2 to 4 µg/mL (MIC_50_ 4 µg/mL, MIC_90_ 4 µg/mL) (Table S1, Supplementary Materials). Notably, NTX also exhibited strong activity against wild-type *E. coli* isolates (*n* = 8), which are resistant to either colistin, meropenem, or tigecycline (Table S3, Supplementary Materials).

To investigate the in vivo therapeutic potential of NTX, we first examined its antibacterial activity under various environmental conditions, including aerobic, microaerophilic, and anaerobic conditions, as well as at temperatures of 37 °C and 42 °C, the latter representing the temperature of the chicken intestine [36]. The results revealed NTX consistently exhibited good potency against *E. coli* and *Salmonella*, with MICs of 2–4 µg/mL (Table S4, Supplementary Materials), regardless of the culture conditions.

### 2.2. In Vitro Resistance Development of NTX

**Figure 2:**
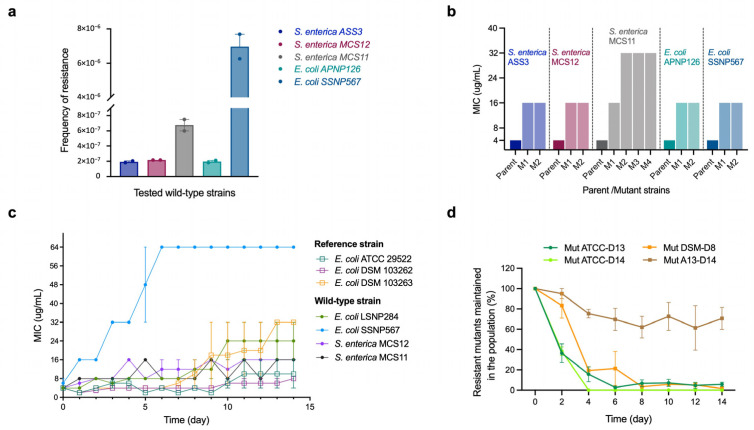
NTX-resistant mutant selection and stability. (**a**) Spontaneous mutation frequency of NTX resistance in wild-type *S. enterica* and *E. coli* strains was measured at 2×, 4×, and 8× MIC (8, 16, and 32 mg/L) after 48 h incubation at 37 °C. The resistance of colonies was supported by their ability to grow on NTX-containing media at the indicated concentrations. The frequency of resistance was determined by dividing the number of resistant mutants by the total number of cells determined by using dilutions of the overnight culture on agar media. Data are presented as mean ± Standard Error of the Mean (SEM) from two technical replicates (*n* = 2). No mutations were observed at the concentration of 4× or 8× MIC (16 and 32 µg/mL, respectively). (**b**) MIC fold changes in selected mutant relative to their parental strains from (**a**). Parent–mutant pairs are indicated by vertical dotted lines. (**c**) Serial passage induction of the resistance to NTX against reference (hollow circle) and wild-type (solid star) *E. coli* and *S. enterica*. The y axis is the MIC-fold change in the tested isolates. Data are presented as mean ± SEM from two biological replicates (*n* = 2). (**d**) Stability of NTX resistance mutations in two mutants from *E. coli* ATCC 25922 (Mut ATCC-D13 and Mut ATCC-D14) and two mutants from *E. coli* DSM 103263 (Mut DSM-D8 and Mut DSM-D14) in the absence of NTX. Mutant stability was quantified as the percentage of mutants in the original bacterial population, calculated by dividing the viable mutant cell count resistant to NTX by the total population and multiplying by a factor of 100. Three biological and two technical replicates were performed for each strain. Data are presented as mean ± SEM (*n* = 3 biological replicates, each with two technical replicates).

To assess the probability of spontaneous resistance to NTX, twenty-five reference strains (Table S2, Supplementary Materials) were tested to determine their frequency of resistance (FoR) at 2×, 4×, and 8× the MIC, with none of the strains producing resistant mutants under their optimal growth conditions. Ten wild-type strains with diverse antimicrobial resistance phenotypes (five *E. coli* and five *S. enterica* isolates; Table S5, Supplementary Materials) were evaluated in FoR analyses. The frequency of spontaneous mutants ranged from 2.0 × 10^−7^ to 7.0 × 10^−6^ for *E. coli* strains and from 1.9 × 10^−7^ to 6.7 × 10^−7^ for *S. enterica* at 2× MIC (8 µg/mL) (Figure 2a; Table S5, Supplementary Materials). However, no mutations were observed at 4× or 8× the MIC in any of the isolates tested (16 and 32 µg/mL, respectively) (Table S5, Supplementary Materials). MICs of NTX-resistant mutants were determined by broth microdilution assays, ranging between 16 and 32 µg/mL (Figure 2b).

To further evaluate the development of NTX resistance, in vitro serial passage studies were conducted. Three reference strains and four wild-type strains of *E. coli* and *S. enterica* were challenged with sub-inhibitory NTX concentrations for 14 consecutive days. Most of the *E. coli* strains developed resistance gradually, with MICs increasing between 4-fold and 16-fold (Figure 2c). The highest MIC of 64 µg/mL was observed with *E. coli* SSNP567 after 6 days. A moderate increase in resistance was observed for both *S. enterica* strains, with MICs rising from 4 to 16 µg/mL after 14 days of NTX exposure (Figure 2c).

To investigate the stability of the NTX-resistant mutants, two mutants from *E. coli* ATCC 25922 (Mut ATCC-D13 and Mut ATCC-D14) and two from *E. coli* DSM 103263 (Mut DSM-D8 and Mut DSM-D14) (Figure 2c,d) were passaged for 14 days in drug-free media. The initial MICs served as benchmarks to evaluate whether resistance persisted in the absence of NTX (Table S6, Supplementary Materials). Mutant stability was quantified as the percentage of mutants in the original bacterial population, calculated by dividing the viable mutant cell count resistant to NTX (8 or 16 µg/mL; Table S6, Supplementary Materials) by the total population and multiplying by a factor of 100. As illustrated in Figure 2d, resistance to NTX in three of the four mutants was unstable, with mutant populations declining from 100% to <20% by day 4. In contrast, resistance in the Mut DSM-D14 strain was partially maintained, with ~70% of the mutant population persisting by day 14. Additionally, MICs of NTX against resistant mutants from *E. coli* ATCC 25922 and DSM 103263 decreased from 16 µg/mL to 4–8 µg/mL and 32 µg/mL to 8–16 µg/mL, respectively, after 14 days without NTX exposure (Table S6, Supplementary Materials).

### 2.3. NTX In Vivo Tolerability and Impact on the Chicken Microbiome

**Figure 3:**
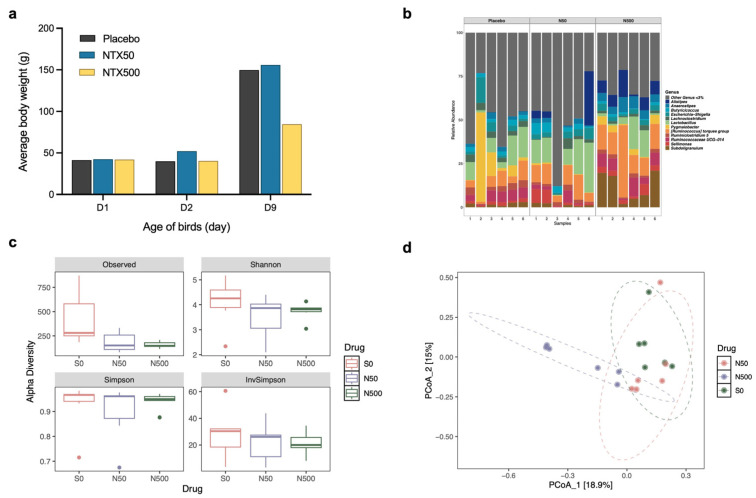
NTX tolerance studies with broilers. (**a**) Average body weight (BW) of broiler chickens fed with 50 ppm (mg/kg) (blue) or 500 ppm (yellow) NTX for 7 days (days 2–9), compared with the placebo (black, non-supplemented basal diet). Data represent the mean BW of birds in each group (*n* = 10). (**b**) Relative abundance of caecal microbiota at the genus level in birds fed with NTX-supplemented diets (50 and 500 ppm) compared to the placebo. Taxa with a relative abundance of <3% were grouped into “Others”. (**c**) Alpha diversity estimates of the bacterial communities in the caecum of chickens treated with NTX at 50 and 500 mg/kg, compared to a placebo. Box plots of bacterial alpha diversity assessed by Shannon, InvSimpson, Choa1, and Observed indexes, and the three lines from bottom to top are: first quartile, median and third quartile. Whiskers extend from the minimum and maximum values, and dots indicate outliers. Statistical differences were determined by ordinary one-way ANOVA with Dunnett’s multiple comparisons test using GraphPad Prism 10.0.3. (**d**) Principal coordinate analysis (PCoA) plot comparing the microbial communities (Bray–Curtis dissimilarity) in cecum of chickens treated with NTX. Six birds were randomly selected per group for (**b**–**d**). The centroid of each ellipse (dotted line) represents the group mean, while the ellipse shape reflects the covariance (dispersion) of samples within each group.

To evaluate the in vivo tolerance of NTX, a preliminary study was conducted in chickens. Dosing was based on that reported for halquinol, given the structural similarities of its components with NTX (Figure 1a), the comparable in vitro activities of NTX and halquinol (Figure 1b), and because halquinol is used as a veterinary antimicrobial feed additive [37,38,39,40,41,42,43]. Halquinol is typically administered orally via feed at inclusion rates ranging from 60 to 600 mg/kg for swine up to 10 consecutive days [37,38,39,40,41,42,43], and 30–1000 mg/kg in broiler chickens up to 42 days [37,38,39,40,41]. Two-day-old chicks were fed diets containing 0 mg/kg (Placebo), 50 mg/kg (NTX50), or 500 mg/kg (NTX500) of NTX (equivalent to ~0, 100, and 1000 mg/kg body weight (BW) per bird per day) for 7 consecutive days. The highest dose tested remained below concentrations associated with adverse effects in acute toxicity studies of halquinol in rats, as reported by the Food and Agriculture Organization (no signs of toxicity were observed at doses of 500 or 1000 mg/kg BW) [36]. On day 9, compared to the placebo groups, birds in the NTX500 group showed a ~43% reduction in average body weight, while those in the NTX50 group exhibited a mild increase in BW (Figure 3a). No abnormal clinical signs were observed. General necropsy examinations (*n* = 6 per group), with particular attention to the heart, liver, spleen, lungs, kidneys, intestines, and yolk sacs, revealed no macroscopic abnormalities.

Differences in haematological parameters were not significant between NTX-treated and the control group (*p* > 0.05; Table S7, Supplementary Materials). Blood biochemical analysis, however, indicated elevated levels of blood urea nitrogen (BUN) and inorganic phosphorus (IP) in the NTX500 group compared to the control group (*p* < 0.05; Figure S1, Supplementary Materials).

To assess the impact of high-dose NTX (500 mg/kg in feed) on the gastrointestinal (GI) microbiota, we performed 16S ribosomal RNA (16S rRNA) gene sequencing of the cecum contents. Relative abundance profiles at the genus level revealed distinct microbial composition differences between NTX-treated and the control groups (Figure 3b). NTX treatment at 500 mg/kg significantly enriched the abundance of *Subdoligranulum* (*p* = 0.0026), *Ruminococcus torques* (*p* = 0.0009), and *Alistipes* (*p* = 0.0396), while reducing *Pygmaiobacter* (*p* = 0.0004). *Escherichia-Shigella* was observed to be lower in the NTX50 (4%) and NTX500 (3%) groups compared to the placebo (5%), but the difference was not significant (*p* > 0.05). *Lactobacillus* and *Butyricicoccus* were increased in NTX50 (14% and 4%, respectively), but decreased in NTX500 (7% and 1%, respectively) compared to the placebo (10% and 2%, respectively).

Microbial diversity was reduced in both the NTX groups, with NTX500 showing significantly lower Chao 1 and Observed indexes compared to controls (*p* < 0.05) (Figure 3c). Principal coordinate analysis (PCoA) based on Bray–Curtis dissimilarity revealed three distinct clusters by treatment, with NTX500 diverging most from the placebo (Figure 3d).

Collectively, these results indicate that NTX alters the caecal microbiome, with a more pronounced effect at the higher NTX concentration (500 mg/kg in feed). Given its in vitro antibacterial activity, tolerability under short-term exposure conditions (despite the reduced BW and altered biochemical parameters at the highest dose tested), and the observed moderate disruption of the gut microbiota, NTX was further examined in in vivo efficacy studies.

### 2.4. NTX Demonstrates In Vivo Antibacterial Activity

To investigate the in vivo antibacterial activity of NTX, we assessed its efficacy in a chicken model infected with wild-type *Salmonella pullorum* (*S. pullorum* SP7) via oral administration. Pullorum disease (PD), caused by *Salmonella enterica serovar* Gallinarum biovar Pullorum, is a poultry-specific infection that leads to high mortality in young birds and reduced productivity, resulting in significant economic losses [44,45]. While largely eradicated in commercial flocks in developed countries, PD is a persistent challenge in China, with frequent outbreaks reported [46,47,48] and was therefore chosen as an infection model. The continued use of antimicrobials to control PD has contributed to the emergence of resistant bacterial strains [47,48]. A preliminary experiment was first conducted to determine the optimal inoculum of the challenge strain *S. pullorum* SP7 to induce mild to moderate symptoms. All animal experiments were approved by the official ethical committees of the University of Oxford, the Biology Animal Welfare and Ethical Review Body (AWERB) [49] (Ref. No.: APA/1/5/ZOO/NASPA/IOI/NTX2022 and APA/1/5/ZOO/NASPA/IOI/NTXII2023), and China Agricultural University (license key: SYXK (Jing) 2018-0038).

**Figure 4:**
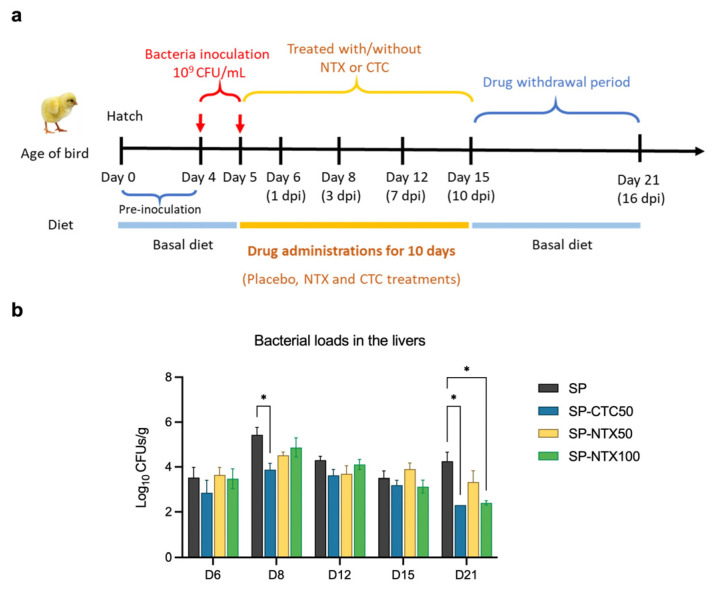
NTX has in vivo antibacterial activity. (**a**) Experimental design of the efficacy study. Birds were given orally the bacteria inoculum (*S. pullorum* SP7, 10^9^ CFU/1 mL) on day 4 and 5 of age, then fed diets containing chlortetracycline (CTC, 50 ppm) or NTX (50 and 100 ppm) for 10 days. dpi: days post-infection. (**b**) Bacterial loads (log CFU/g) in livers. Placebo: non-infected, untreated; SP: infected, untreated; SP-CTC50: infected, treated with 50 ppm CTC; SP-NTX50/100: infected, treated with 50 or 100 ppm NTX. Bars represent mean ± SEM (*n* = 6). Statistical differences were determined by two-way ANOVA with Dunnett’s multiple comparisons test using GraphPad Prism 10.0.3. Asterisks denote significant differences compared to SP group (*, *p* < 0.05).

The results indicated that an oral inoculum of 10^9^ colony-forming units (CFUs)/mL administered on days 4 and 5 resulted in a moderate BW loss (11%) compared to non-infected birds, when measured at day 10 (5 days post-infection; dpi) (Figure S2a, Supplementary Materials). No notable clinical symptoms or mortalities were observed. Gross necropsy and microbiology testing supported *S. pullorum* colonisation in the liver and revealed lesions in the kidney, spleen, and intestines (Figure S2b, Supplementary Materials). By contrast, groups receiving 10^7^ or 10^8^ CFU/mL inoculum exhibited less pronounced clinical signs than the 10^9^ CFU/mL group. Higher inoculum doses and earlier inoculation (prior to day 4) were not examined in compliance with the UK policy on the use of animals in scientific research [49]. To evaluate the efficacy of NTX, four-day-old chicks were orally infected with *S. pullorum* for 2 consecutive days, followed by a ten-day treatment with NTX at 50 mg/kg or 100 mg/kg, or with chlortetracycline (CTC) at 50 mg/kg [50] (Figure 4a,b). The selected NTX dosages (50 and 100 mg/kg) were based on findings from the preliminary safety study, which indicated that higher doses (500 mg/kg) induced reduced BW and markedly altered the chicken’s microbiome (Figure 3). Viable *S. pullorum* bacteria were identified in liver samples from infected birds in all groups beginning at 1 dpi (day 6) (Figure 4b; Figure S3a, Supplementary Materials). By day 8 (after 3 days of treatment), the bacterial loads in the liver were reduced in treated groups compared to the untreated infection control group (Figure 4b). Although NTX at 100 mg/kg demonstrated slower initial efficacy compared to CTC, it achieved comparable antibacterial efficacy with CTC by the end of the treatment period (day 21). After discontinuing treatment for 6 days following the end of the trial (day 21; 16 dpi), sustained inhibitory effects on *S. pullorum* invasion were observed in the NTX100 group. This equates to an ~270-fold reduction in bacterial loads (2.7 × 10^2^ CFU/g, *p* < 0.05) and a significantly lower detection rate (33.3%, *p* < 0.05) compared to the *S. pullorum* group (7.3 × 10^4^ CFU/g; 83.3%) (Figure 4b; Figure S3a, Supplementary Materials).

In vivo resistance was evaluated by measuring MICs of NTX in *S. pullorum* isolates (*n* = 3 per group) from infected livers after 10 days of treatment (Figure S3b, Supplementary Materials). No significant changes were observed, suggesting substantial in vivo resistance to NTX is not present under these experimental conditions.

### 2.5. Pharmacokinetics Profiles and Tissue Distribution of NTX in Chickens

**Figure 5:**
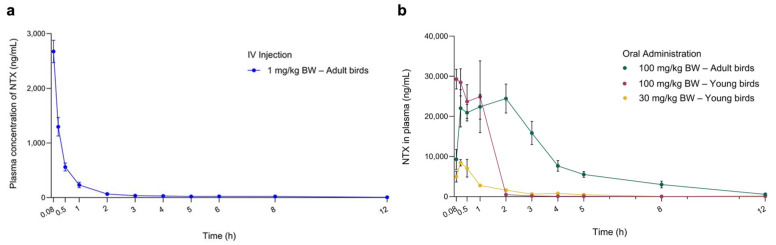
Plasma concentration-time curves of NTX determined in chickens following a single intravenous (IV) injection (**a**) and oral administration (**b**) at the indicated doses. Data are presented as mean ± SEM (*n* = 7) for adult birds receiving 100 mg/kg NTX; *n* = 6 for all other groups. NTX was not detected at 24, 36 and 48 h in plasma; therefore, only data up to 12 h are presented.

Pharmacokinetic (PK) studies were conducted to characterise the plasma profiles of NTX in adult chickens (forty-two-day-old) following a single oral dose (PO; 100 mg/kg BW) or intravenous injection (IV; 1 mg/kg BW). NTX appeared rapidly in the bloodstream after oral administration, reaching a peak concentration (C_max_ = 32,257.29 ± 9265.32 ng/mL) at T_max_ = 1.36 ± 1.02 h, with a moderate half-life (T_1/2_ = 6.51 ± 2.03 h) (Figure 5a, Table 1). These results suggest that NTX undergoes rapid absorption, distribution, and elimination following oral administration. The absolute oral bioavailability (F_PO_) of NTX in adult birds was calculated to be 86%.

Given the F_PO_ and in vivo efficacy data, five-day-old birds received single oral doses of NTX at 100 mg/kg BW and 30 mg/kg BW to further assess plasma PK profiles. The corresponding concentration-time curves are shown in Figure 5b, and primary PK parameters obtained through non-compartmental analysis are presented in Table 1. At 100 mg/kg BW, NTX was detected in plasma for 2 h in young birds (five-day-old) and for more than 5 h in adult chickens (forty-two-day-old), maintaining concentrations above the MIC of 4 µg/mL required for *S. pullorum* inhibition (Figure 5b; Figure S3b, Supplementary Materials). This sustained plasma level may contribute to the observed in vivo antibacterial activity of NTX (Figure 4b). Birds administrated 100 mg/kg BW NTX exhibited a significantly higher area under the curve (AUC) and C_max_, as well as a lower mean residence time (MRT_last_), compared to those receiving 30 mg/kg BW (*p* < 0.01) (Figure 5a, Table 1).

**Figure 6:**
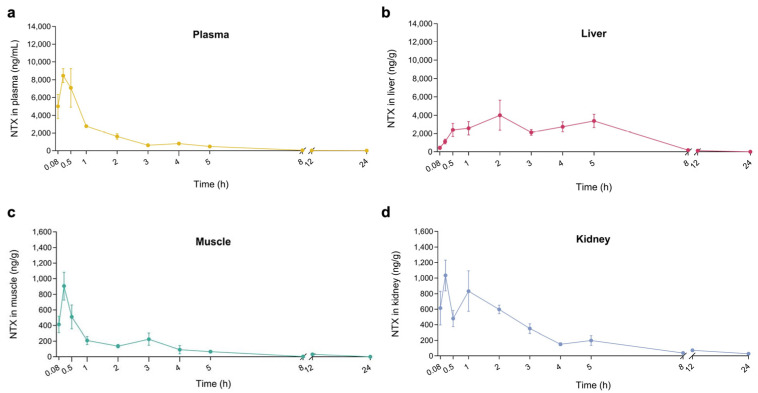
NTX concentration-time curves in plasma (**a**), liver (**b**), muscle (**c**) and kidney (**d**) following a single oral administration at 30 mg/kg BW. Data are presented as mean ± SEM (*n* = 6). NTX was not detected at 36 and 48 h in plasma and tissues; therefore, only data up to 24 h are presented.

To evaluate NTX tissue distribution, we measured NTX concentrations in liver, kidney, and muscle samples following a single oral dose of 30 mg/kg BW. NTX was detected in all three tissues within five minutes, with peak concentrations observed at 2 h in the liver and at 0.25 h in the kidney and muscle (Figure 6b–d). These findings indicate rapid NTX absorption from the GI tract and rapid tissue distribution. Notably, the highest NTX concentrations were found in the liver (4004 ng/g), followed by kidney (1035 ng/g) and muscle (905 ng/g) samples (Figure 6b–d; Table S9, Supplementary Materials). Overall, these results suggest that NTX is rapidly absorbed, extensively distributed throughout the body of the chicken, and exhibits a moderate half-life with high bioavailability.

### 2.6. Residue Clearance of NTX in Chicken Tissues

**Figure 7:**
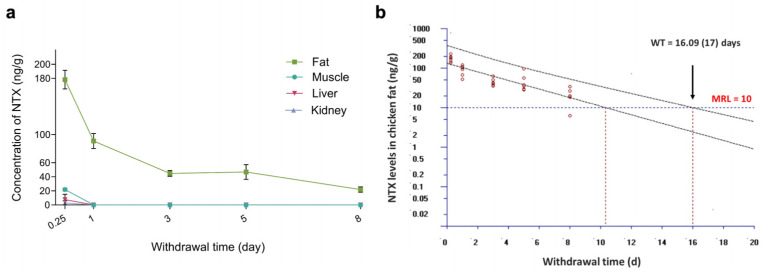
Residue clearance of NTX in chicken tissues following withdrawal of feed containing 100 mg/kg NTX for 10 consecutive days. Data are presented as mean ± SEM (*n* = 6). (**a**) Clearance profiles of NTX in chicken skin-plus-fat (green), muscle (blue), liver (red) and kidney (purple). (**b**) Plot of withdrawal time (WT) calculation for NTX in chicken skin-plus-fat, based on the time at which the one-sided 95% upper tolerance limit fell below the Korean MRL for NTX (10 ng/g) [54]. Each red circle represents an individual measured concentration of NTX at a given sampling time. The black lines are the linear regression and 95% tolerance limit with 95% confidence. The blue horizontal line denotes the MRL (10 ng/g).

To determine NTX concentrations in tissues following 10 consecutive days of administration via the feed, muscle, liver, kidney, and skin-plus-fat samples were collected from thirty healthy NTX-treated birds at 6 h, and at 1, 3, 5, and 8 days after the final treatment (six birds per time point). Notably, NTX exhibited rapid elimination from the liver and kidney, with only one chicken having tissue samples above the corresponding limit of quantification (LOQ, 10 ng/g) at 6 h (Figure 7a; Table S10, Supplementary Materials). The NTX level in muscle was 21.96 ± 5.06 ng/g at 6 h post-withdrawal and fell below the LOQ within 1 day after the final dose (Figure 7a; Table S10, Supplementary Materials). In contrast, NTX selectively accumulated in skin-plus-fat, with the highest residue level (178.03 ng/g) being detected at 6 h post-withdrawal, and levels remained above the LOQ for up to 8 days after cessation of administration (Figure 7a). Using the withdrawal time (WT) calculation program WT 1.4, the estimated WT for skin-plus-fat was determined to be 17 days (Figure 7b). As NTX is not approved for use in poultry production in the European Union (EU) or US (United States), no established tolerance or maximum residue limit (MRL) currently exists for chickens treated with NTX [51,52,53]. Therefore, the WT estimated in this study—calculated using the Korean poultry MRL (10 ng/g) [54]—should be considered provisional and subject to future jurisdiction-dependent regulations. Because NTX concentrations were only quantifiable at the first post-withdrawal time point (Table S10, Supplementary Materials), WT values for muscle, liver, and kidney could not be determined.

### 2.7. Impact on Growth Performance of Broilers

**Figure 8:**
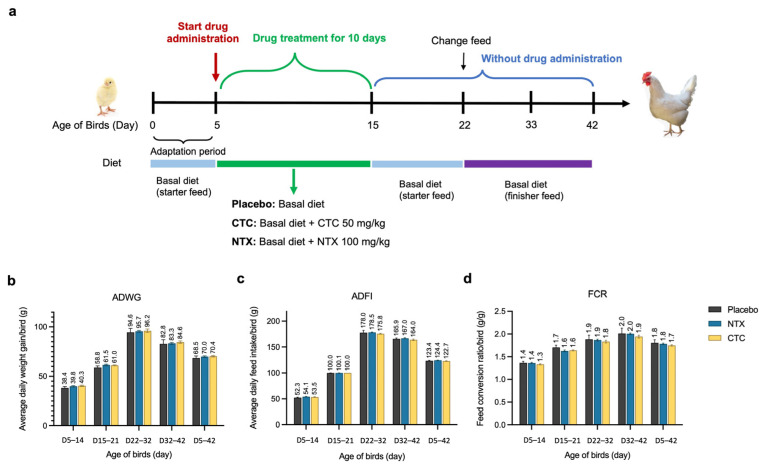
Effect of NTX on growth performance of broiler chickens. (**a**) Schematic representation of the experimental design. Average daily weight gain (ADWG) (**b**), average daily feed intake (ADFI) (**c**), or the feed conversion ratio (FCR) (**d**) in different treatment groups. Data are presented as the mean ± SEM (*n* = 12).

To investigate the impact of NTX on broiler growth performance, we conducted experiments with three test groups receiving dietary treatments: NTX (100 mg/kg), CTC (50 mg/kg), and a placebo control. These groups were based on data from the in vivo efficacy studies. Treatments were administered continuously for 10 days, from days 5 to 15 (Figure 8a). Throughout the feeding phases, all chickens remained healthy, and no significant differences were observed among the three groups for average daily weight gain (ADWG), average daily feed intake (ADFI), or the feed conversion ratio (FCR) (*p* > 0.5). During the ten-day treatment period (D5–14), NTX and CTC supplements numerically improved ADWG by 3.7% and 4.8%, and ADFI by 3.5% and 2.3%, respectively, compared to the placebo. Feed efficiency, as indicated by FCR, was improved in both groups, with NTX and CTC reducing FCR by 1.0% and 2.4%, respectively (Figure 8b–d). In the subsequent post-treatment phase (D15–21), NTX and CTC groups maintained higher ADWG (4.6% and 3.9% greater than the control) and lower FCR (4.3% and 3.8% lower than the control) (Figure 8b–d). Over the complete 42-day experimental period (D5–42), NTX supplementation resulted in daily weight gain comparable to CTC (2.1% and 2.7% higher than the control, respectively). However, while the CTC group consumed less feed and exhibited a 3.2% lower FCR than the control, the NTX group showed a 1.3% lower FCR. These results indicate that NTX provides weight gain and feed efficiency benefits comparable to those of CTC. Additionally, carcass traits were evaluated at day 42, with the highest carcass yield at 91%, semi-eviscerated rate at 85%, and fully eviscerated rate at 74% observed in NTX-treated birds; however, no significant differences were detected among groups (Figure S4, Supplementary Materials).

### 2.8. Impact on Caecal Microbiota of Broilers

**Figure 9:**
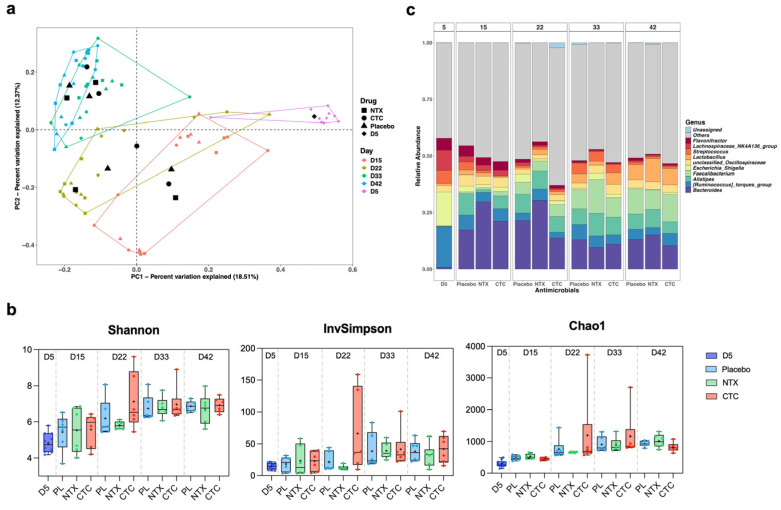
Effect of NTX on caecal microbiome of broiler chickens. Birds were treated with either NTX (100 mg/kg) or CTC (50 mg/kg) continuously for 10 days (days 5–15) and compared with a placebo group (fed a non-supplemented basal diet). 16S ribosomal RNA (rRNA) amplicon sequencing was performed on caecal contents collected on day 5 (*n* = 10 total) and on days 15, 22, 33, and 42 from six randomly selected broilers per group (*n* = 1 per replicate). (**a**) Principal coordinate analysis (PCoA) plot of microbial communities in the caecum at different ages. Analysis was based on Bray–Curtis dissimilarity; samples were grouped according to age. The centroid of each ellipse represents the group mean. (**b**) Boxplots of alpha diversity as measured by Shannon, InvSimpson, and Chao1 diversity indexes. The box extends from the 25th to 75th percentiles; the index values are presented as the median (central black horizontal line); the whiskers extend from the minimum and maximum values. + represents the mean values of the replicates for each group (*n* = 10 for day 5, *n* = 6 per group for all other days). Data from different sampling days are separated by vertical dotted lines. Statistical differences were determined by ordinary one-way ANOVA with Dunnett’s multiple comparisons test using GraphPad Prism 10.0.3. (**c**) Average relative abundances of microbial community in the cecum at the genus level. Taxa with a relative abundance of <1% were grouped into “Others”.

To further investigate the impact of NTX on the GI microbiota of broilers, 16S rRNA sequencing was performed on caecal contents collected on days 5, 15, 22, 33, and 42 (*n* = 10 for day 5; *n* = 6 per group thereafter) (Figure 8a). PCoA based on Bray–Curtis dissimilarity showed clear age-related clustering, with high variability at day 5 and increased similarity by days 33 and 42, regardless of treatment (Figure 9a). Microbial diversity (Shannon, InvSimpson, and Chao1) increased significantly with age, peaking at days 22 and 33, then stabilising (Figure 9b). At day 22, diversity—reflected by evenness (InvSimpon) and richness (Chao1)—rose in CTC-treated birds, but apparently declined in NTX-treated birds compared to the placebo, though the differences were not significant (*p* > 0.05; Figure 9b).

Relative abundance profiles revealed distinct age-dependent shifts in the caecal microbiome (Figure S6, Supplementary Materials; Figure 9c). Firmicutes, Bacteroidota, and Proteobacteria were dominant, with Firmicutes decreasing from day 5 to 22 before rising to day 42, and Bacteroidota increasing until day 33, then remaining relatively stable thereafter (Figure S6, Supplementary Materials). At day 22, NTX-treated birds showed higher Firmicutes and Bacteroidota levels compared to controls (*p* > 0.05), whereas CTC treatment significantly reduced Bacteroidota (*p* < 0.05). By day 42, Firmicutes in NTX-treated birds dropped significantly to 70% (*p* < 0.005), while Bacteroidota rose to 24% (*p* < 0.05) compared to controls, which were 78% and 20%, respectively.

At the genus level, *Bacteroides* and *Alistipes* (Bacteroidota), *Faecalibacterium* and *Lactobacillus* (Firmicutes) exhibited similar increasing trends as their corresponding phyla (Figure 9c). In contrast, *Escherichia-Shigella* (Proteobacteria) and *Ruminococcus torques* (Firmicutes) declined in all groups from day 5 onwards. By day 15, *Alistipes* abundance decreased in drug-treated birds (NTX: 1.2%, *p* = 0.0014; CTC: 6.1%, *p* > 0.05) compared to placebo treatment (9.7%), but gradually recovered after drug withdrawal (Figure S8, Supplementary Materials). Bacteroides, the most predominant genus, peaked in NTX-treated birds and declined significantly in CTC-treated birds by day 22 (*p* < 0.05) (Figures S7 and S8, Supplementary Materials). By day 33, *Faecalibacterium* was significantly more abundant in the NTX group (14%) than in the control group (8%) (*p* < 0.5). Notably, *Lactobacillus* increased in all groups by day 42, with the highest level in NTX (10%), followed by CTC (7%) and placebo (7%), though differences were not significant (Figure 9c; Figures S7 and S8, Supplementary Materials).

To assess the potential for NTX-induced resistance in the GI microbiota, caecal contents were plated on NTX-supplemented agar (2, 4, and 8 µg/mL). Bacterial growth on day 5 was used as a baseline for subsequent resistance assessment. No resistant bacterial colonies were observed at 8 µg/mL at any time point (Figure S5, Supplementary Materials), indicating a low in vivo resistance risk for NTX.

## 3. Discussion

NTX has been used to treat UTIs since 1962 [55]. We selected NTX as a candidate for use in livestock farming due to its broad antibacterial spectrum, simple structure/low manufacturing cost, and low toxicity in clinical studies [56,57]. Various studies have reported on the activity of NTX against clinical bacterial strains and even against MDR pathogens [23,26,58,59]. To explore whether NTX could be used to replace widely used human antibiotics (e.g., aminopenicillins, tetracyclines, quinolones), which are currently extensively used in animals, we first examined NTX against eighty-eight wild-type poultry isolates (Table S1, Supplementary Materials), including drug-resistant pathogens (Table S3, Supplementary Materials); the resultant MICs ranged from 2 to 4 µg/mL.

Importantly, the emergence of NTX resistance occurred with a relatively low frequency, consistent with previous findings [32,33,60]. In FoR studies with wild-type *E. coli* and *S. enterica* isolates (Table S5, Supplementary Materials), NTX-insusceptible mutants occurred at 2× MIC NTX (8 µg/mL), reflecting in low-level resistance (a 4-fold to 8-fold MIC shift) (Figure 2b). Notably, no mutants were observed when selected on media containing NTX at 4× or 8× MIC, indicating that physiological barriers limit the development of higher levels of NTX resistance, as observed in previous studies [32,33]. Furthermore, NTX mutants generated by passaging were shown to be unstable and/or imposed a fitness cost (Figure 2d), as noted in previous studies [32]. Importantly, when we examined the development of in vivo resistance, *S. pullorum* colonies isolated from the livers of NTX-treated chickens showed no resistance to NTX, as evidenced by the unaltered MICs (Figure S3b, Supplementary Materials). Although genomic characterization of NTX-resistant mutants was beyond the scope of the present study, reported work indicates that NTX resistance primarily involves mutations in genes regulating efflux systems (including EmrAB–TolC and RND families), membrane permeability, and metal homeostasis, consistent with NTX’s proposed multi-target, metal-chelating mechanism of action [32,33,60].

In vivo antibacterial analysis with NTX administration in feed at 100 mg/kg reduced *Salmonella* loads in the liver, leading to an approximately 270-fold decrease in bacterial loads (*p* < 0.05) (Figure 4b) an amount comparable with CTC, which is widely used to treat *Salmonella* infections in poultry [50,61]. Our preliminary safety assessment of NTX in chickens (10-day tolerability, necropsy, haematological and biochemical analyses and impact on intestinal microflora) showed no clinical contraindications for NTX even when administered at high doses (Figure 3).

From a regulatory perspective, the present findings represent a proof-of-concept rather than a comprehensive safety assessment. Authorization of veterinary medicinal products or feed additives for use in food-producing animals requires extensive toxicological evaluation, including acute toxicity, short-term toxicity, long-term toxicity and carcinogenicity, genotoxicity, reproductive and developmental toxicity studies, as mandated by regulatory authorities. In the EU, the legal framework for the approval and monitoring of veterinary medicinal products is set by Regulation (EU) 2019/6, with guidelines from the European Medicines Agency (EMA)/Committee for Veterinary Medicinal Products (CVMP) [62,63]. Similar oversight is provided by the US Food and Drug Administration’s Center for Veterinary Medicine (FDA-CVM) (which requires a New Animal Drug Application for such approvals) and the Joint FAO/WHO Expert Committee on Food Additives (JECFA) [52,64]. While our short-term tolerability data suggest that NTX is well tolerated under the tested conditions, the observed reduction in BW (Figure 3a) and changes in biochemical parameters at high doses (Figure S1, Supplementary Materials) indicate that further long-term and dose-ranging studies are required prior to regulatory approval.

The residual level of antibiotics potentially present in retail meat is a health concern for consumers [65]. When we administered NTX at 100 mg/kg (in feed), the longest residual time was for skin-plus-fat compared to other tissues (muscle, liver, and kidney), with residue levels above the LOQ (10 ng/g) 8 days after drug withdrawal (Figure 7a; Table S10, Supplementary Materials). According to the MRL of NTX from Korea (10 ng/g) [54], the WT for NTX in skin-plus-fat is 17 days in broilers (Figure 7b). Our results show that NTX is eliminated quickly from the liver, kidney, and muscle tissues, but more slowly from fat; however, at the time of slaughter (42 days), levels of NTX are predicted to be <1 ng/g.

At present, no internationally harmonized MRLs for NTX have been established by Codex Alimentarius or other international regulatory authorities [51,52,53]. Consequently, residue compliance requirements for NTX may differ substantially between jurisdictions, something that has important implications for regulatory approval and international trade of poultry products. The reliance on a national MRL in this study highlights the need for comprehensive residue metabolism studies, including characterization of NTX metabolites, to support global regulatory evaluation and future harmonization of MRLs prior to broader commercial deployment [53,54,55,56,57,58,59,60,61,62,64].

A key parameter for any food additive is whether it adversely affects growth, as this can have an economic bearing on profit margins, with the risk of exacerbating poverty in low- and middle-income countries (LMICs) [65,66,67]. We therefore calculated the ADWG, ADFI and FCR values for different phases (Figure 8), comparing NTX to CTC. The results show that the ADWG at day 42 was improved by 2.1% in the NTX group, and 2.7% in the CTC group, compared to the control. These results suggest a potential improvement in nutrient absorption, supporting the economic case for NTX.

From an economic perspective, NTX is a small-molecule compound with a simple and well-established synthetic route (Figure 1), suggesting that its large-scale manufacturing costs may be comparable to or lower than those of many currently used veterinary antimicrobials. While a detailed cost-effectiveness analysis was beyond the scope of this study, the comparable growth performance observed between NTX and CTC (Figure 8) supports the potential economic feasibility of NTX as an alternative antimicrobial in poultry production.

We examined the impact of NTX administration and its withdrawal on the intestinal microbial community of broilers. Several bacterial taxa, particularly those within the genera *Lactobacillus* and *Faecalibacterium*, are generally considered to be beneficial for host intestinal heath [68]. In our study, the relative abundance of *Lactobacillus* showed a slight decrease in the NTX-administered group at day 15, followed by a gradual increase, reaching its highest level by day 42, with lower levels observed in the CTC and placebo groups (Figure S8, Supplementary Materials). *Faecalibacterium*, a genus associated with the production of butyrate and other short-chain fatty acids in the host gut [69], showed a significant increase with NTX at day 33 (Figure S8, Supplementary Materials), further supporting the hypothesis that NTX modulates caecal bacterial composition.

NTX contains a nitro group—the debate on the safety of these compounds in farmed meat and human consumption ensues and needs to be addressed [70]. Currently, the guidance for long-term toxicity studies for compounds used in food-producing animals lacks standardisation; for example, food safety studies on halquinol (which is structurally related to NTX, but which does not contain a nitro group) vary significantly [71,72,73]. If we aspire to have sustainable food production whilst concurrently addressing the UNGA HLM targets on antibiotic use in agriculture, there needs to be a consensus on how new compounds/therapies are evaluated with respect to human food safety [64]. In the case of antibiotics containing nitro groups, long-term (one year) safety experiments, where higher animals (e.g., dogs) are given cooked chicken meat (where the chickens are exposed to regular doses of new compounds/therapies) would provide additional important safety data.

Importantly, authorization of NTX for use in poultry production will require compliance with established veterinary medicinal product or feed additive approval frameworks. In the EU, this would involve assessment by the EMA through the CVMP, including evaluation of efficacy, target animal safety, consumer safety (toxicology, residue depletion, establishment of MRLs and WTs), and environmental risks [62,63]. Comparable requirements apply in non-EU jurisdictions, including the US FDA-CVM [52] and JECFA-guided [64] frameworks in other regions, although specific regulatory pathways may differ by jurisdiction.

Accordingly, the findings presented here should be interpreted as an experimental proof-of-concept demonstrating antimicrobial efficacy, pharmacokinetics, residue depletion characteristics, and short-term tolerability of NTX in poultry. This study does not constitute regulatory authorization for use as a veterinary medicinal product or feed additive in food-producing animals. Transition from experimental evaluation to authorized use will require additional standardized regulatory studies, including long-term toxicology, residue metabolism, and environmental impact assessments, conducted in accordance with region-specific regulatory guidelines [51,52,53,62,63].

Whilst our study provides a detailed in vitro and in vivo assessment of NTX for use in poultry, it has several limitations. Firstly, we used *S. pullorum* for efficacy studies, which is an invasive model and not necessarily relevant to countries outside Southeast (SE) Asia; therefore, additional infection models, e.g., *Salmonella enteritidis* and/or Avian Pathogenic *E. coli* (APEC), will need to be undertaken. Secondly, although we recognise that NTX will be relatively cost-effective to produce, we did not undertake a full economic assessment of its manufacturing costs and the current agriculture market competitors, e.g., CTC, ampicillin, and colistin [74]. Thirdly, for the drug residue analysis, we examined NTX but not its metabolites. Although we would expect the metabolites to be rapidly excreted, this should be addressed in future regulatory-compliance residue metabolism studies.

The injudicious use of “human antibiotics” in animal husbandry/farming is now in sharp focus following the agreement on UNGA HLM targets [14]. The agricultural use of colistin selecting for *mcr-*1 (and related variants), and oxytetracycline/demethylchlortetracycline/CTC selecting for *tet*(X2) to *tet*(X15) and mediating tigecycline resistance, act as a warning in deploying the same compounds or class for both human medicine and food-producing animals [75,76]. Currently, there exists a human-only antibiotic list [77], but the animal-only antibiotic recommendation category is still being debated [78]. Given the above, we must be more imaginative and consider how we can replace the widely used and vital human antibiotics currently deployed in animal production and so mitigate cross-resistance to human pathogens.

## 4. Materials and Methods

### 4.1. Bacterial Strains and Growth Conditions

The reference bacterial strains used for preliminary screening are listed in Table S2 (Supplementary Materials) and were from commercial suppliers. Wild-type poultry-sourced *S. enterica* and *E. coli* strains (Tables S3 and S5, Supplementary Materials) were sourced from laboratory collections. The wild-type *S. pullorum* strain (SP7) used in in vivo efficacy studies was isolated from a poultry farm in China and maintained by the College of Veterinary Medicine, China Agricultural University (Beijing, China). All strains were identified by Matrix-assisted laser desorption ionization–time of flight (MALDI-TOF) mass spectrometry (Bruker Daltonics, Bremen, Germany). Culture conditions and media requirements for growth are detailed in Table S2 (Supplementary Materials).

### 4.2. Chemical and Reagents

All compounds and solvents were from Sigma-Aldrich (St. Louis, MO, USA), Fluorochem Ltd. (Derbyshire, UK), Tokyo Chemical Industries (Tokyo, Japan), or MedChemExpress (Monmouth Junction, NJ, USA), and used without further purification. Stock solutions were prepared in dimethyl sulfoxide (DMSO) at 2560 µg/mL and stored at −20 °C. NTX (purity > 99.57%) and CTC (purity > 95.0%) powder for in vivo studies were obtained from MedChemExpress (Monmouth Junction, NJ, USA). Analytical-grade NTX standards (purity > 99.9%) for high performance liquid chromatography (HPLC) analysis were sourced from Tianjin Alta Scientific Co., Ltd. (Tianjin, China). HPLC-grade solvents (acetonitrile, methanol, formic acid, isopropanol, ammonium hydroxide) were purchased from Thermo Fisher Scientific (Waltham, MA, USA). All other reagents used in the PK and drug residue studies were of analytical grade. Ultrahigh purified water was produced using a Milli-Q water purification system (Merck Millipore, Burlington, MA, USA).

### 4.3. In Vitro Susceptibility Testing

MICs were determined using the broth microdilution method following the CLSI guidelines [35]. Test compounds were two-fold serially diluted (0.0625–64 µg/mL) in a 96-well microtiter plate. Bacterial suspensions were adjusted to 0.5 McFarland standard (corresponding to approximately 10^8^ CFU/mL) by adding sterile broth and were added to each well. Plates were incubated under strain-specific conditions (Table S2, Supplementary Materials). Compounds with MIC ≤ 16 µg/mL were selected for further testing under varied growth conditions, including aerobic, microaerophilic, and anaerobic environments at 37 °C and 42 °C. MIC was defined as the lowest concentration inhibiting visible bacterial growth. All assays were performed in duplicate.

### 4.4. In Vitro Resistance Development Studies

Spontaneous resistance frequency was determined by plating 0.1 mL of overnight cultures on Mueller-Hinton agar (MHA) containing NTX at 2×, 4×, and 8× MIC (8, 16, and 32 mg/L). Plates were incubated aerobically at 37 °C for 24–48 h. Resistant colonies were confirmed by growth on NTX-containing media (2× or 4× MIC) and quantified as FoR, calculated as the ratio of resistant colonies to total viable cells. Confirmed mutants were re-streaked on NTX-supplemented agar at the same concentration used for selection, grown in liquid culture, and stored at −80 °C. Two technical repeats were performed per strain.

For sequential passaging, exponential-phase bacterial cultures (approximately 10^6^ CFU/mL) were exposed to a range of NTX concentrations in 96-well plates and incubated at 37 °C for 18–24 h. MICs were defined as the lowest concentration preventing visible growth. Cultures from sub-MIC wells were diluted 1000-fold into fresh NTX-containing medium daily for 14 days. Control cultures were passaged without NTX. Two biological replicates were conducted per strain.

### 4.5. Stability of NTX Resistance Mutations

Four NTX-resistant *E. coli* mutants (Mut ATCC-D13, Mut ATCC-D14; Mut DSM-D8, Mut DSM-D14) from fourteen-day serial passage were cultured daily for 14 days in antibiotics-free fresh medium (1:1000 dilution) at 37 °C, 200 rpm. On days 0, 2, 4, 6, 8, 10, 12 and 14, aliquots were plated onto CAMHB agar with or without NTX (2× or 4× MIC). Resistance was assessed by bacterial growth on NTX-containing medium (2× or 4× MIC). Mutant stability was quantified as the percentage of NTX-resistant cells in the original bacterial population, calculated by dividing the viable mutant cell count resistant to NTX (2× or 4× MIC) by the total population and multiplying by a factor of 100. Each strain was tested in triplicate with two technical repeats. Parental strains *E. coli* ATCC 25922 and *E. coli* DSM 103263 were patched on drug-free medium only as controls. MICs of four colonies per strain were determined at day 14 using broth microdilution.

### 4.6. Ethical Statements and Animal Experiments

Healthy commercial Arbor Acres (AA) broiler chickens were obtained from the Zhuozhou Teaching Experimental Base of China Agricultural University (Zhuozhou, China). Birds were allocated to experimental floor pens in an environmentally controlled broiler house, designed to maintain optimal temperature, humidity, lighting, ventilation, and feed conditions [79]. Chicks underwent an acclimation period prior to experimentation, with ad libitum access to water and feed provided via an automatic feeding system, including height-adjustable drinking channels and floor feeders to accommodate different growth stages. The basal diet used consisted of a two-phase commercial corn and soybean meal-based diet (starter and finisher), formulated to meet broiler nutritional requirements for market growth and free of antimicrobial additives [79]. Stock density was maintained at an average of <12 kg/m^2^, considerably below the UK recommended threshold of 25 kg/m^2^ for meat chickens [80].

Birds were monitored four times daily by trained technicians for clinical signs of illness or discomfort, including weakness, loss of appetite, poor growth (12.5% lower weight gain vs. non-infected birds) [80,81], and abnormal droppings. Feed intake (FI) and BW were recorded at appropriate intervals. Birds showing moderate disease symptoms were removed for sedation and humane euthanasia. However, no euthanasia was required in this study, as infection induced only mild symptoms (moderate BW reduction) (Figure S2a, Supplementary Materials). When organ examinations were needed, birds were humanely sacrificed by individually removing the chickens to a sawdust-floored chamber and gradually sedated with CO_2_ inhalation followed by cervical dislocation [82].

### 4.7. Investigation of NTX Tolerance in Chickens

Forty AA broilers (one-day-old) were randomly assigned to three groups (*n* = 10): (1) Placebo, receiving a non-supplemented, antibiotic-free diet; (2) NTX50, receiving 50 ppm (mg/kg) NTX; (3) NTX500, receiving 500 ppm NTX. NTX was administered by mixing it with feed for 7 consecutive days, from day 2 to day 9 of age. Birds had ad libitum access to feed and water. At day 10, blood (*n* = 3) and caecal (*n* = 6) samples were collected from each group for subsequent haematology, blood chemistry, and microbiota analysis. Non-toxic concentrations of NTX were used for further experiments.

### 4.8. Haematology and Blood Biochemistry Analysis

Blood samples were collected on day 10 at the end of the tolerance study (*n* = 3 per group). Whole blood (1 mL) was drawn from the right jugular vein into dipotassium ethylenediaminetetraacetic acid (K_2_ EDTA) tubes (BD Bioscience, San Jose, CA, USA) for haematology, and 2 mL was collected in serum separator tubes (SST, BD Bioscience, San Jose, CA, USA) for blood biochemistry analysis. Haematological parameters included red blood cells (RBC), haemoglobin (HGB), haematocrit (HCT), mean corpuscular volume (MCV), mean corpuscular haemoglobin concentration (MCHC), white blood cells (WBC), neutrophils (Neu), lymphocytes (Lym), monocytes (Mon), eosinophils (Eos), basophils (Bas), platelets (PLT), and mean platelet volume (MPV). For serum biochemistry, blood samples were left at room temperature for at least 30 min, then centrifuged at 2000 rpm at 4 °C for 10 min to separate the serum. Biochemical parameters included total protein (TP), albumin (ALB), globulins (GLB), BUN, creatinine (CRE), creatine kinase (CK), calcium (Ca), IP.

### 4.9. In Vivo Efficacy Study Using a Salmonella Infection Chicken Model

The optimal challenge dose was established through preliminary experiments assessing infection intervals (once on day 5 of age or twice on days 4 and 5 of age) and doses (10^7^, 10^8^, and 10^9^ CFU/1 mL per chicks) (Figure S2, Supplementary Materials), identifying 10^9^ CFU/mL of *S. pullorum* SP7 on days 4 and 5 as optimal, inducing moderate BW reduction (11%) without minor clinical signs, and clear liver colonisation and lesions in the kidney, spleen, and intestines (Figure S2, Supplementary Materials).

Bacteria inocula were prepared by culturing *S. pullorum* SP7 in Luria–Bertani (LB) broth and incubating at 37 °C with shaking at 150 rpm for 24 h until reaching the mid-logarithmic phase of growth. The culture was then centrifuged at 8500 rpm for 5 min, washed, and resuspended in sterile LB to obtain a final bacterial density of 10^9^ CFU/mL.

A total of two hundred sixteen healthy AA broiler chicks (one-day-old) were randomly assigned to six treatment groups (*n* = 36/group; three replicates of twelve birds): (1) placebo (uninfected, untreated); (2) SP (infected, untreated); (3) SP-CTC50 (infected, treated with 50 ppm CTC); (4) SP-NTX50 (infected, treated with 50 ppm NTX); (5) SP-NTX100 (infected, treated with 100 ppm NTX). Infected groups received oral *S. pullorum* SP7 (10^9^ CFU/mL) on days 4 and 5 of age, while the placebo group received sterile LB broth in a similar manner (Figure 4a). The birds were fasted for 4 h prior to infection. NTX or CTC was administered via feed from days 5 to 15 (10 consecutive days).

Birds were monitored regularly for clinical signs and mortalities throughout the experimental period. Clinical signs, including mental status, coat condition, behaviour changes, faecal characteristics, morbidity, and mortality were recorded. Symptoms were categorised by severity: mild (reduced FI, cold sensitivity, body curled up, drooping wings, depression), moderate (white sticky or light yellow/green loose stools, sometimes obstructed anus with hardened faecal mass, along with mild symptoms); and severe (no FI, breathing difficulties, and the presence of severe symptoms) [83].

The primary efficacy endpoint was bacterial load in the liver, determined by CFU counts on agar plate as described by Xu et al. [83]. Livers were collected from six birds/group at days 6 (1 dpi), 8 (3 dpi), 12 (7 dpi), 15 (10 dpi), and 21 (16 dpi) (Figure 4a), homogenized in sterile PBS, serially diluted, and plated on MacConkey agar (Remel, Lenexa, KS, USA), and incubated at 37 °C for 24 h to determine bacterial load (CFU/g liver). Liver samples from two birds/group were screened prior to infection to confirm absence of *S. pullorum*.

### 4.10. In Vivo Assessment of NTX Resistance Development

To evaluate potential resistance acquisition by *S. pullorum*, infected chickens were treated with NTX (50 or 100 ppm) for 10 days (Figure 4a). MICs of NTX were determined for randomly selected *S. pullorum* isolates obtained from the livers of birds in the SP-NTX50 and SP-NTX100 groups. On day 15, two isolates per treatment group were recovered from individual livers and subjected to MIC testing via the microbroth dilution method, with two technical replicates per isolate.

In a separate 42-day growth performance study (Figure 8a), NTX-induced resistance in chicken intestinal flora, particularly among Enterobacteriaceae, was evaluated after 10 days of drug administration. Caecal samples were collected on days 5 (pre-treatment), 15, 22, 33, and 42 (*n* = 2/replicate; *n* = 6/group). Samples were processed and plated on chromogenic UTI agar supplemented with vancomycin (10 µg/mL; to suppress Gram-positive bacteria) and either NTX (2, 4, or 8 µg/mL) or CTC (4, 8, 16, or 32 µg/mL); plates with vancomycin alone served as controls. NTX (8 µg/mL) and CTC (32 µg/mL) were selected as reference concentrations for resistance monitoring based on baseline growth (day 5). Caecal contents were suspended in sterile saline, filtered, and appropriately diluted. A 100 µL aliquot of suspensions was spread onto the corresponding drug-containing or drug-free plates in duplicate. Suspensions from the NTX group were plated on NTX and drug-free plates, while those from the CTC group were plated on CTC and drug-free plates. Suspensions from the placebo group were spread on NTX, CTC, and drug-free plates as controls. Plates were incubated at 37 °C for 24–48 h, and bacterial growth was observed and recorded. Isolates were identified using MALDI-TOF mass spectrometry.

### 4.11. In Vivo Pharmacokinetics Study

Thirteen healthy six-week-old adult AA broilers were randomly assigned to two groups. One group (*n* = 7) received a single oral dose of NTX (100 mg/kg BW), while the other (*n* = 6) received a single IV dose (1 mg/kg BW) via the brachial wing vein. Additionally, 160 healthy one-week-old broilers were divided into two groups (*n* = 72) and received a single oral dose of NTX at 100 mg/kg BW or 30 mg/kg BW. Blood samples (0.3–0.5 mL) were collected from the medial metatarsal vein (adult birds) or right jugular vein (young birds) at the following time points: 0.083, 0.25, 0.5, 1, 2, 3, 4, 5, 6, 8, 12, 24, 36 and 48 h post-administration (*n* = 6 birds at each sampling point). Samples were centrifuged at 4000 rpm for 10 min at 4 °C to obtain plasma. For young birds receiving a single oral dose of 30 mg/kg BW, breast muscle, liver (entire organ) and kidney (entire organ) were collected separately at the corresponding time points to investigate tissue distribution. NTX concentrations in plasma and tissue samples were analysed by ultra-performance liquid chromatography–tandem mass spectrometry (UPLC-MS/MS). PK parameters in plasma were calculated using a non-compartmental model in Phoenix WinNonLin software (version 6.4.0, Pharsight Corporation, Mountain View, CA, USA). Oral bioavailability (%) was calculated using the following equation: F_PO_ (%) = 100 × (AUC_last,PO_/AUC_last,IV_) × (Dose_IV_/Dose_PO_), where AUC_last_ represents AUC from the time of dosing to the last measurable concentration following PO and IV administration, respectively.

### 4.12. NTX Residue in Chicken Tissues

Thirty healthy five-day-old AA broiler chickens were administered NTX at 100 ppm via feed for 10 consecutive days. Tissue samples–including breast muscle, liver (entire organ), kidney (entire organ) and fat/skin–were collected separately at 0.25, 1, 3, 5 and 8 days post-administration (*n* = 6 birds per time point). Homogenized tissues were extracted twice with acetonitrile (5–7 mL), purified using commercial solid-phase extraction (SPE) cartridges (MedChem Express, Monmouth Junction, NJ, USA), and analysed for NTX residues using UPLC-MS/MS. The WT was estimated using linear regression of a semi-logarithmic tissue concentration plot, with the upper one-sided 95% tolerance limit falling below the MRL [63]. In the absence of an established MRL for NTX in the US and EU, Korea’s poultry MRL (10 ng/g) was used to calculate the WT [54]. Statistical analysis and visualization were performed using the WT calculation program WT 1.4 [84].

### 4.13. Growth Performance and Carcass Traits of Broilers

A total of two hundred seventy healthy one-day-old AA broiler chickens were randomly assigned to three treatment groups (*n* = 90/group, six replicates of fifteen birds): (1) placebo, basal diet; (2) NTX, basal diet supplemented with 100 ppm NTX; and (3) CTC, basal diet supplemented with 50 ppm CTC. The 42-day experiment consisted of an initial basal diet phase (days 1–4), a medication period (days 5–15), and a drug withdrawal phase (days 16–42) (Figure 8a). Chicks received antibiotic-free starter feed until day 21, then transitioned to finisher feed. Feed and antibiotic-free water were available ad libitum throughout the study.

BW and FI per replicate were recorded on days 5, 15, 22, 33, and 42. Growth performance parameters–including ADWG, ADFI, and FCR (calculated as FI divided by WG)–were calculated accordingly.

At the end of the trial (day 42), twelve birds per treatment group (*n* = 2/replicate) were randomly selected for carcass evaluation. Live weight (LW) was recorded before slaughter. Carcass weight (CW) was recorded after defeathering, with the head and feet included. Semi-eviscerated weight (SEW) was determined as CW minus the trachea, esophagus, GI tract, crop, spleen, pancreas, gallbladder, and gonads. Full eviscerated weight (FEW) was recorded after further removal of the head, feet, heart, liver, gizzard, glandular stomach, and abdominal fat. Carcass yield, semi-eviscerated yield and full eviscerated yield were calculated as the percentage of LW.

### 4.14. Caecal Microbiota Analysis

rRNA amplicon 16S sequencing was performed on caecal contents collected at multiple time points. In the tolerance study, samples were taken on day 10 (*n* = 6/group); in the growth performance study, samples were obtained on day 5 (*n* = 10 total), 15, 22, 32, and 42 (*n* = 6/group, one bird per replicate).

Genomic DNA was extracted from 0.2 g of caecal contents using ZymoBIOMICS DNA Microprep Kit (Zymo Research, Irvine, CA, USA). DNA quantity and quality were measured using agarose gel electrophoresis and a Tecan infinite F200 fluorescence microplate reader (Tecan Group AG, Männedorf, Switzerland). Amplicons from the V3-V4 regions of the 16S rRNA gene were generated from the extracted DNA using primers 341F (5′-CCTACGGGRSGCAGCAG-3′) and 806R (5′-GGACTACHVGGGTWTCTAAT-3′), and polymerase chain reaction (PCR) clean-up was performed using Zymoclean Gel Recovery Kit (Zymo Research, Irvine, CA, USA). NEBNext Ultra II DNA Library Prep Kit (NEW ENGLAND BioLabs, Ipswich, MA, USA) was used to generate Illumina libraries, and sequencing was performed using the Illumina MiSeq platform (NovaSeq 6000 Reagent Kits, Illumina, San Diego, CA, USA) generating paired-end 250 bp reads.

For the microbiota analysis, the Quantitative Insights into Microbial Ecology (QIIME 2) bioinformatics platform (https://qiime2.org/) (accessed on 14 July 2025) was used [85]. Quality control of the raw reads was performed using FastQC 0.11.8 (Babraham Bioinformatics: https://www.bioinformatics.babraham.ac.uk/projects/fastqc/) (accessed on 14 July 2025). Trimmomatic 0.33 was used to trim the adapter and other illumina-specific sequences (http://www.usadellab.org/cms/?page=trimmomatic) (accessed on 14 July 2025). The trimmed sequences (fastq.gz) were imported into QIIME 2 as a manifest file format (PairedEndManifestPhred33V2). A feature table was constructed, and additional filtering of the sequences was performed using DADA2 [86]. The taxonomic analysis was performed using Naive Bayes classifiers trained on the Silva 138 database. The phylogenetic diversity was analysed using the align-to-tree-mafft-fasttree pipeline, and alpha and beta diversity were analysed using the core metrics-phylogenetic pipeline (https://docs.qiime2.org/2019.7/tutorials/moving-pictures/) (accessed on 14 July 2025) [87].

### 4.15. Statistical Analyses

Statistical comparison between groups was performed using GraphPad Prism 10.0.3., with methods specified in the figure legends. A *p* value < 0.05 was considered statistically significant.

## Figures and Tables

**Table 1:** Key pharmacokinetic parameters of NTX in chickens following a single intravenous injection or oral administration at the indicated doses.

Parameter	Unit	IV Injection 1 mg/kg BW (Adult Birds, *n* = 7)		① PO 100 mg/kg BW (Adult Birds, *n* = 6)		② PO 100 mg/kg BW (Young Birds, *n* = 6)		③ PO 30 mg/kg BW (Young Birds, *n* = 6)		*p* Value (① vs. ②)	*p* Value (② vs. ③)
		Mean	SD	Mean	SD	Mean	SD	Mean	SD		
λ_z_	1/h	0.25	0.24	0.12	0.04	0.077	0.023	0.09	0.04	0.0778	0.4200
T_1/2_	h	5.19	3.09	6.51	2.03	9.507	2.670	10.94	10.12	0.1937	0.7421
T_max_	h	0.08	0.00	1.36	1.02	0.360	0.349	0.29	0.10	0.0603	0.6934
C_max_	ng/mL	2675.50	505.81	32,257.29	9265.32	37,590.83	12,177.39	10,051.83	4176.31	0.4649	0.0030 **
AUC_last_	h × ng/mL	1245.97	266.69	106,548.69	15,081.42	38,588.19	13,853.40	12,037.07	2555.14	0.0006 ***	0.0074 **
AUCINF__obs_	h × ng/mL	1273.46	259.78	107,007.49	15,616.70	38,919.96	13,985.43	12,361.08	2441.27	0.0007 ***	0.0075 **
AUC__%Extrap_obs_	%	2.37	1.96	0.38	0.73	0.798	1.073	2.78	1.94	0.5470	0.0849
MRT_last_	h	2.23	1.26	3.97	2.16	1.677	0.726	3.55	0.94	0.0513	0.0077 **

IV, intravenous injection; PO, oral administration; λ_z_, elimination rate constant; T_1⁄2_, terminal elimination half-life; T_max_, time to the maximum concentration; C_max_, maximum concentration; AUC_last_, Area Under the Curve (AUC) from the time of dosing to the last measurable positive concentration; AUCINF__obs_, AUC from dosing time extrapolated to infinity, based on the last observed concentration; AUC__%Extrap_obs_, percentage of AUCINF__obs_ due to extrapolation from T_last_ (last time point with measurable concentration) to infinity; MRT_last_, mean residence time from the time of dosing to the time of the last measurable concentration. Statistical differences were determined by paired t-test using GraphPad Prism 10.0.3. ** *p* < 0.01, *** *p* < 0.001.

## Data Availability

All data are included in this study either in the manuscript or in the Supplementary Materials. Genome sequences can be found under NCBI BioProject ID (PRJNA1274427). Source data are provided with this paper.

## Supplementary Materials

The following supporting information can be downloaded at: https://www.mdpi.com/article/10.3390/antibiotics15010062/s1, Figure S1. Blood biochemical parameters of broiler chickens; Figure S2. Chicken model of *Salmonella* infection; Figure S3. NTX demonstrates in vivo antibacterial activity; Figure S4. Effect of dietary NTX supplementation on carcass traits of broiler chickens at 42 days of age; Figure S5. NTX is unlikely to induce antibiotic resistance in the intestinal microbiota of broiler chickens; Figure S6. Average relative abundances of the microbial community in the cecum at the phylum level; Figure S7. Average relative abundances of the microbial community in the cecum at the genus level on different sampling days; Figure S8. Average relative abundances of the predominant genus in the microbial community in the cecum; Table S1. List of screened compounds and MIX under 37 °C; Table S2. List of pathogenic bacterial strains and culture conditions used in this study; Table S3. NTX activity against wild-type drug-resistant *E. coli* isolates with specific resistance genes; Table S4. Antibacterial activity of NTX against reference *E. coli* and *Salmonella* spp. under 37 °C and 42 °C; Table S5. The wild-type isolates screened for NTX-resistant mutants; Table S6. MICs of NTX for parental strains and mutants on day 0 and day 14; Table S7. Hematologic parameters in broiler chickens after fed with 50 ppm and 500 ppm NTX for 7 consecutive days; Table S8. NTX concentrations in plasma of adult chickens following a single oral administration at 100 mg/kg BW; Table S9. NTX concentrations in plasma and tissues of young chicks following a single oral administration at 30 mg/kg BW; Table S10. NTX residue (ng/g) in chicken tissues following withdrawal of feed containing 100 mg/kg NTX for 10 consecutive days.

## Abbreviations

The following abbreviations are used in this manuscript:

NTX	Nitroxoline
AMU	Antimicrobial use
UNGA HLM	United Nations General Assembly High-Level Meeting
AMR	Antimicrobial resistance
WASH	Water, sanitation and hygiene
MICs	Minimum inhibitory concentrations
MDR	Multidrug-resistant
UTIs	Urinary tract infections
EUCAST	European Committee on Antimicrobial Susceptibility Testing
CLSI	Clinical and Laboratory Standards Institute broth protocol
SAR	Structure-activity relationship
FoR	Frequency of resistance
SEM	Standard error of the mean
BW	Body weight
PCoA	Principal coordinate analysis
GI	Gastrointestinal
rRNA	Ribosomal RNA
*S. pullorum*	*Salmonella pullorum*
*S. enterica*	*Salmonella enterica*
PD	Pullorum disease
CFUs	Colony-forming units
dpi	Days post-infection
CTC	Chlortetracycline
PK	Pharmacokinetic
PO	Oral administration (per os)
IV	Intravenous injection
F_PO_	Oral bioavailability
λ_z_	Elimination rate constant
T_1⁄2_	Terminal elimination half-life
T_max_	Time to the maximum concentration
C_max_	Maximum concentration
AUC	Area under the curve
AUC_last_	AUC from the time of dosing to the last measurable positive concentration
AUCINF__obs_	AUC from dosing time extrapolated to infinity, based on the last observed concentration
AUC__%Extrap_obs_	Percentage of AUCINF__obs_ due to extrapolation from T_last_ (last time point with measurable concentration) to infinity
MRT_last_	Mean residence time from the time of dosing to the time of the last measurable concentration
EU	European Union
US	United States
JECFA	Joint FAO/WHO Expert Committee on Food Additives
FDA-CVM	Food and Drug Administration’s Center for Veterinary Medicine
EMA	European Medicines Agency
CVMP	Committee for Veterinary Medicinal Products
LOQ	Limit of quantification
WT	Withdrawal time
MRL	Maximum residue limit
ADWG	Average daily weight gain
ADFI	Average daily feed intake
FCR	Feed conversion ratio
LMICs	Low- and middle-income countries
SE	Southeast
APEC	Avian Pathogenic *E. coli*
MALDI-TOF	Matrix-assisted laser desorption ionization–time of flight
MHA	Mueller-Hinton agar
AA	Arbor Acres
FI	Feed intake
K_2_ EDTA	Dipotassium ethylenediaminetetraacetic acid
RBC	Red blood cells
HGB	Haemoglobin
HCT	Haematocrit
MCV	Mean corpuscular volume
MCHC	Mean corpuscular haemoglobin concentration
WBC	White blood cells
Neu	Neutrophils
Lym	Lymphocytes
Mon	Monocytes
Eos	Eosinophils
Bas	Basophils
PLT	Platelets
MPV	Mean platelet volume
TP	Total protein
ALB	Albumin
GLB	Globulins
BUN	Blood urea nitrogen
CRE	Creatinine
CK	Creatine kinase
Ca	Calcium
IP	Inorganic phosphorus
LB	Luria–Bertani
UPLC-MS/MS	Ultra-performance liquid chromatography–tandem mass spectrometry
SPE	Solid-phase extraction
LW	Live weight
CW	Carcass weight
SEW	Semi-eviscerated weight
FEW	Full eviscerated weight
PCR	Polymerase chain reaction
QIIME	Quantitative insights into microbial ecology
