# Supplementary material for: In Vitro and In Vivo Evaluation of Nitroxoline as an Effective Antimicrobial Alternative to Poultry Production
Source: Antibiotics (Basel). 2026 Jan 6;15(1):62. doi: 10.3390/antibiotics15010062 (PMC12838059; doi:10.3390/antibiotics15010062)
Supplement: Supplementary file 1 [file antibiotics-15-00062-s001.zip › Supplementary Materials-Revised.pdf]

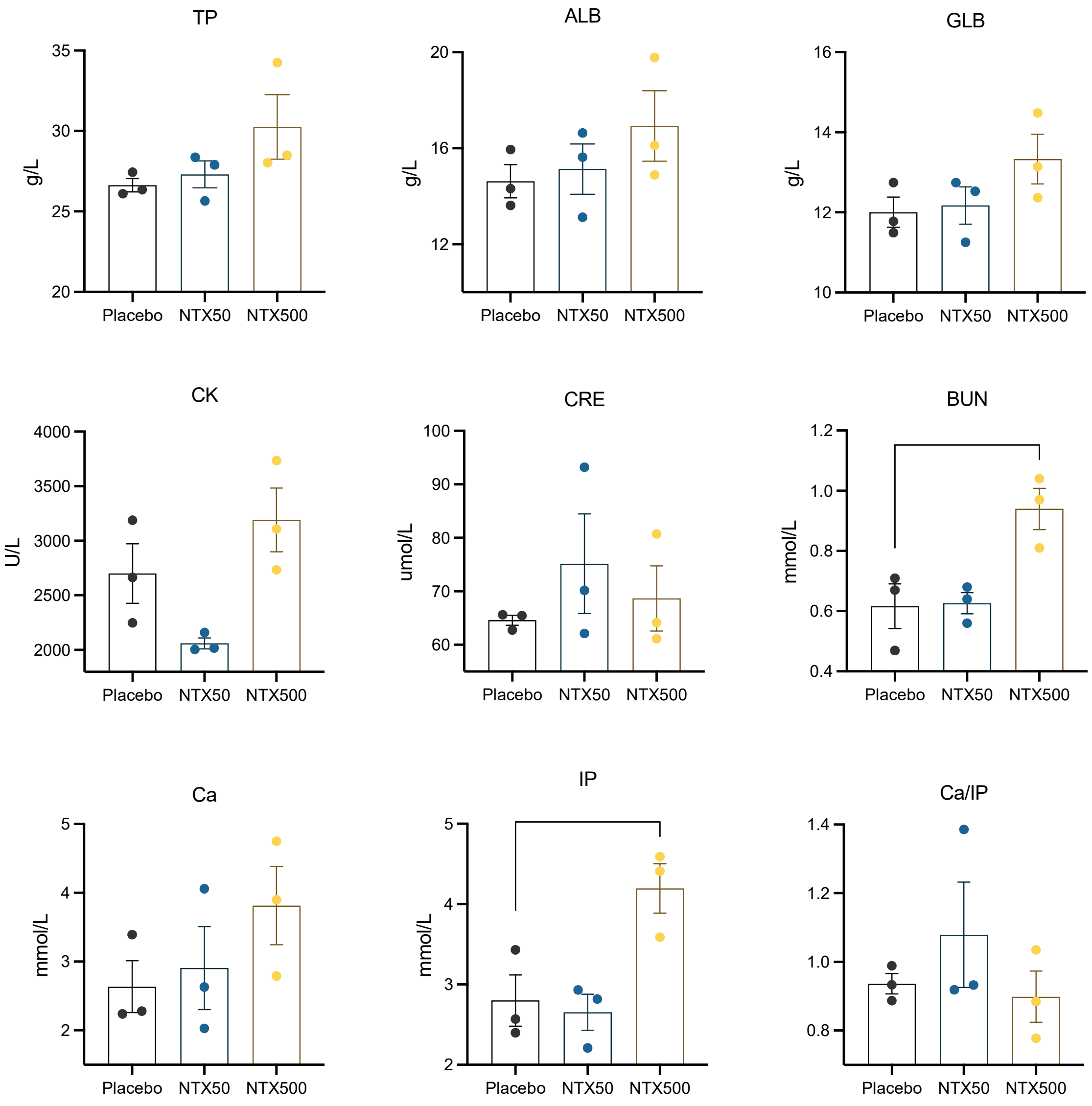

Supplementary Figure S1. Blood biochemical parameters of boiler chickens received feed supplemented with 50 ppm (light blue) and 500 ppm (cream) of NTX for 7 consecutive days, compared with Placebo group (light grey). Total Protein (TP), Albumin (ALB), Globulins (GLB), Creatine Kinase (CK), Creatinine (CRE), Blood Urea Nitrogen (BUN), Calcium (Ca), Inorganic Phosphorus (IP). Three birds were randomly selected from each group. The data are expressed as mean ± SEM (n = 3). Statistical differences were determined by ordinary one-way ANOVA with Dunnett's multiple comparisons test using GraphPad Prism 10.0.3. Asterisks denote significant differences compared to the placebo group (*, $p < 0.05$).

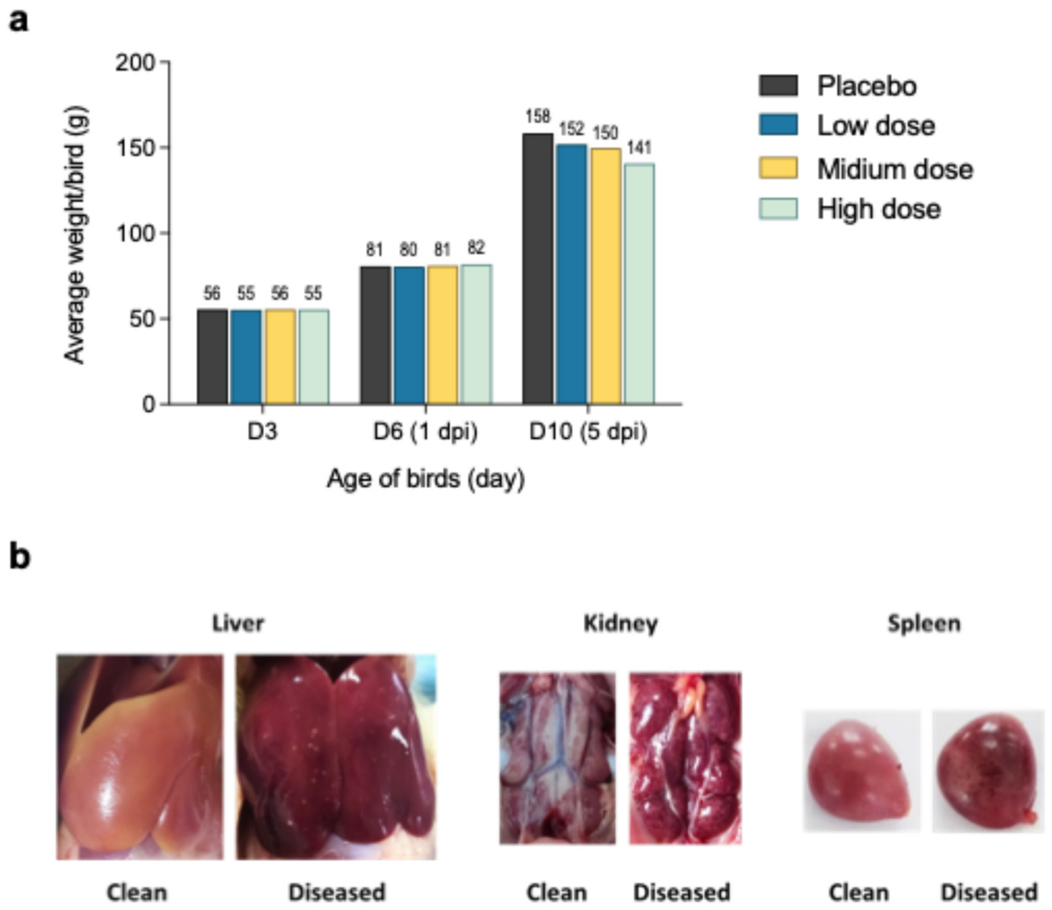

Supplementary Figure S2. Chicken model of *Salmonella* infection. (**a**) Decreased average body weight in chickens infected with a low (10^7 CFU/mL), medium (10^8 CFU/mL) or high (10^9 CFU/mL) dose of *S. pullorum* for 2 consecutive days (days 4 and 5). dpi: days post-infection. Placebo group, non-infected broiler chickens orally administered 1 mL of sterile broth. Data are presented as mean (*n* = 12, per group). (**b**) Representative images of pathological changes in the liver, kidney, and spleen of infected chickens on day 10 (5 dpi) following high-dose *S. pullorum* infection, showing tissue discoloration, enlargement, and necrotic lesions.

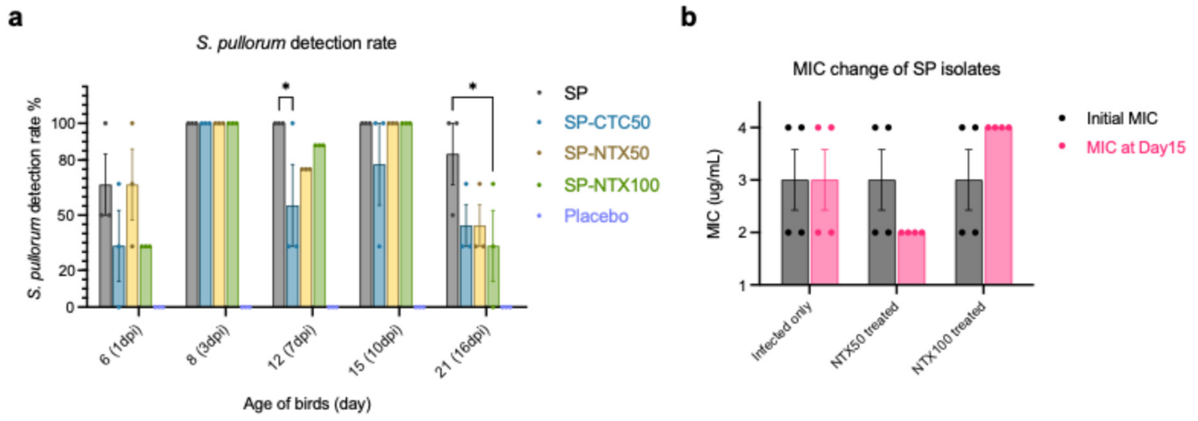

Supplementary Figure S3. NTX demonstrates *in-vivo* antibacterial activity. (**a**) NTX effect on *S. pullorum* (SP) detection rate (%) of *Salmonella*-infected chickens at different time points. dpi: days post-infection. Birds were given orally bacteria inoculum (*S. pullorum* SP7, 10^9 CFU/mL) on day 4 and day 5 of age, then fed diets containing chlortetracycline (CTC, 50 ppm) or NTX (50 and 100 ppm) for 10 days. Placebo: non-infected, untreated; SP: infected, untreated; SP-CTC50: infected, treated with 50 ppm CTC; SP-NTX50/100: infected, treated with 50 or 100 ppm NTX. Data are represented as the mean ± SEM (n = 6). Statistical differences were determined by two-way ANOVA with Dunnett's multiple comparisons test using GraphPad Prism 10.0.3. Asterisks denote significant differences compared to the SP group (*, $p < 0.05$). (**b**) Sensitivity of *S. pullorum* isolates to NTX on day 15, following 10 days of NTX administration at varying doses. Groups: Infected only–birds infected with SP but untreated; NTX50/100 treated–infected birds treated with 50 or 100 ppm NTX diets. Two strains isolated from the livers of two individual birds were selected per group, with two technical replicates per strain. Data represent group mean ± SEM (n = 4).

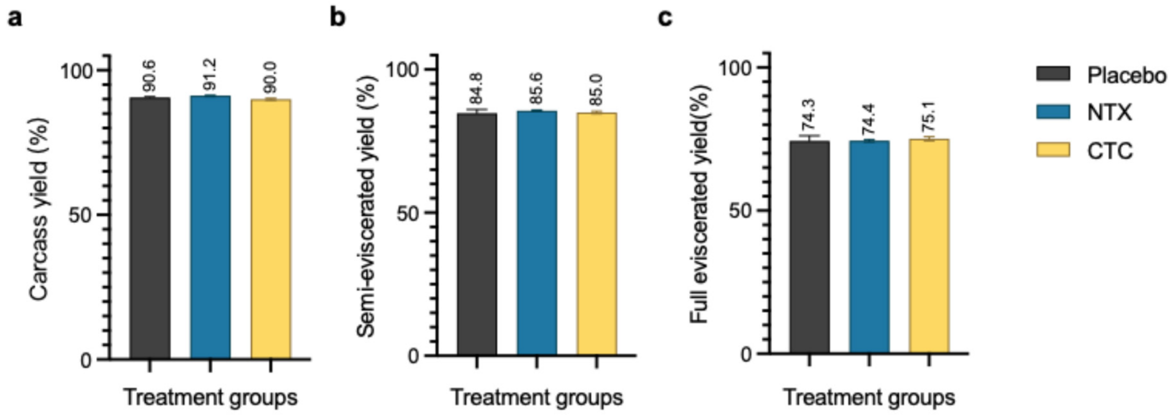

Supplementary Figure S4. Effect of dietary NTX supplementation on carcass traits of broiler chickens at 42 days of age. Carcass yield (**a**), semi-eviscerated yield (**b**) and full eviscerated yield (**c**) in different treatment groups. Chickens received dietary supplements of NTX (100 mg/kg), CTC (50 mg/kg), or a placebo control continuously for 10 days (days 5–15). Data are represented as mean ± SEM (*n* = 12).

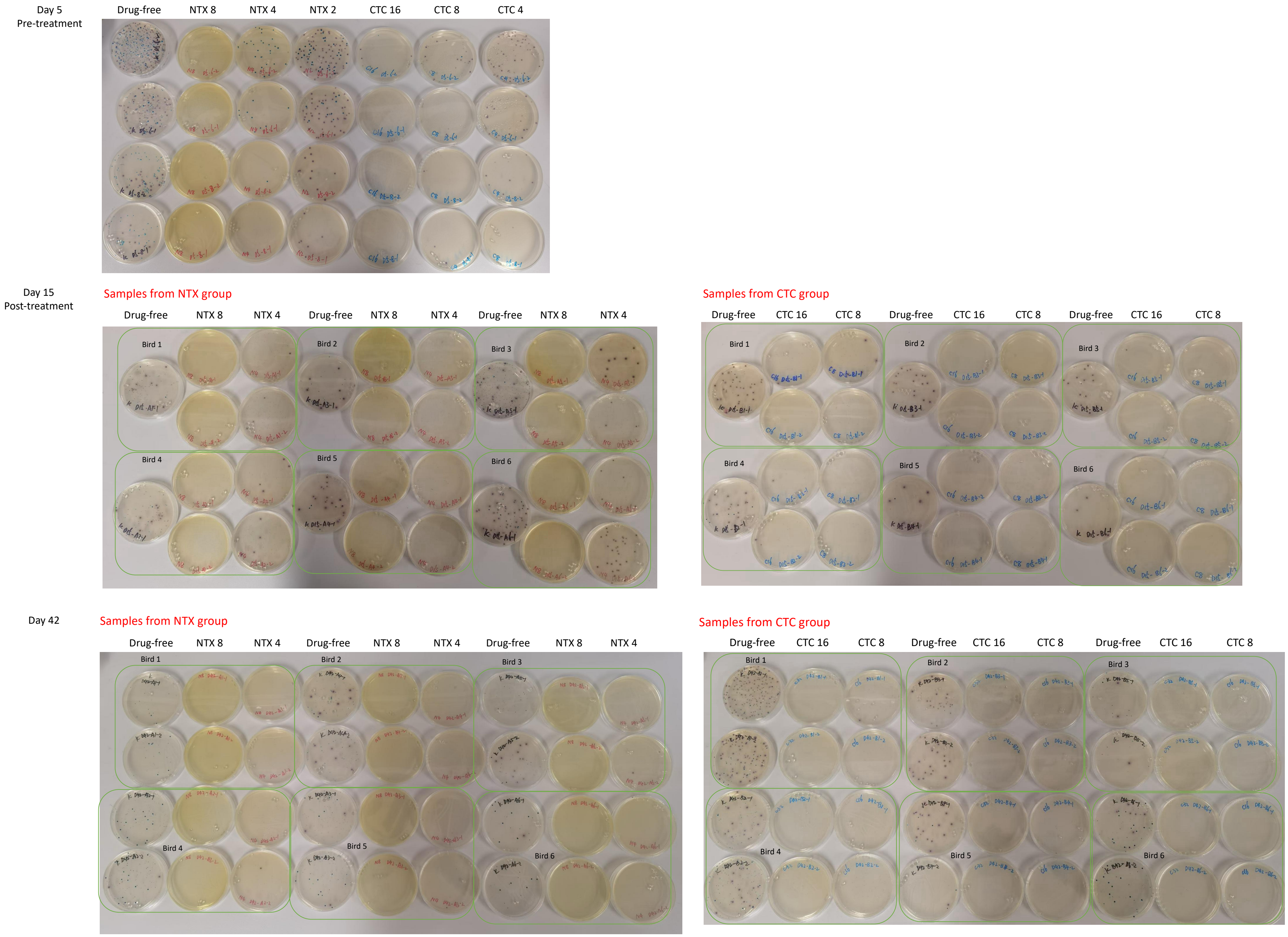

Day 5
Pre-treatment
Drug-free
NTX 8
NTX 4
NTX 2
CTC 16
CTC 8
CTC 4
Day 15
Post-treatment
Samples from NTX group
Drug-free
NTX 8
NTX 4
Drug-free
NTX 8
NTX 4
Drug-free
NTX 8
NTX 4
Bird 1
Bird 2
Bird 3
Bird 4
Bird 5
Bird 6
Samples from CTC group
Drug-free
CTC 16
CTC 8
Drug-free
CTC 16
CTC 8
Drug-free
CTC 16
CTC 8
Bird 1
Bird 2
Bird 3
Bird 4
Bird 5
Bird 6
Day 42
Samples from NTX group
Drug-free
NTX 8
NTX 4
Drug-free
NTX 8
NTX 4
Drug-free
NTX 8
NTX 4
Bird 1
Bird 2
Bird 3
Bird 4
Bird 5
Bird 6
Samples from CTC group
Drug-free
CTC 16
CTC 8
Drug-free
CTC 16
CTC 8
Drug-free
CTC 16
CTC 8
Bird 1
Bird 2
Bird 3
Bird 4
Bird 5
Bird 6

Supplementary Figure S5. NTX is unlikely to induce antibiotic resistance in the intestinal microbiota of broiler chickens**.** The potential for NTX-induced resistance in the chicken intestinal flora, particularly among Enterobacteriaceae, was evaluated following a 10-day drug administration. Caecal samples were collected on days 5 (pre-treatment), 15 (10 days post-treatment), 22, 33, and 42 (*n* = 2 per replicate, *n* = 6 per group). Samples were plated onto agar containing NTX (2, 4, and 8 μg/mL), CTC (4, 8, 16, and 32 μg/mL), or vancomycin alone, which served as a control medium free of NTX and CTC. Based on bacterial growth observed on day 5, NTX concentration of 8 μg/mL and CTC concentration of 32 μg/mL were selected as baselines for subsequent resistance monitoring. No bacterial colonies were observed on plates containing 8 μg/mL NTX at any time point. As a control, caecal contents from the CTC group were plated on agar containing CTC at 8 and 16 μg/mL. Pink and blue colonies were identified as *E. coli* and *K. pneumoniae*, respectively, using matrix-assisted laser desorption ionization–time of flight (MALDI-TOF) mass spectrometry.

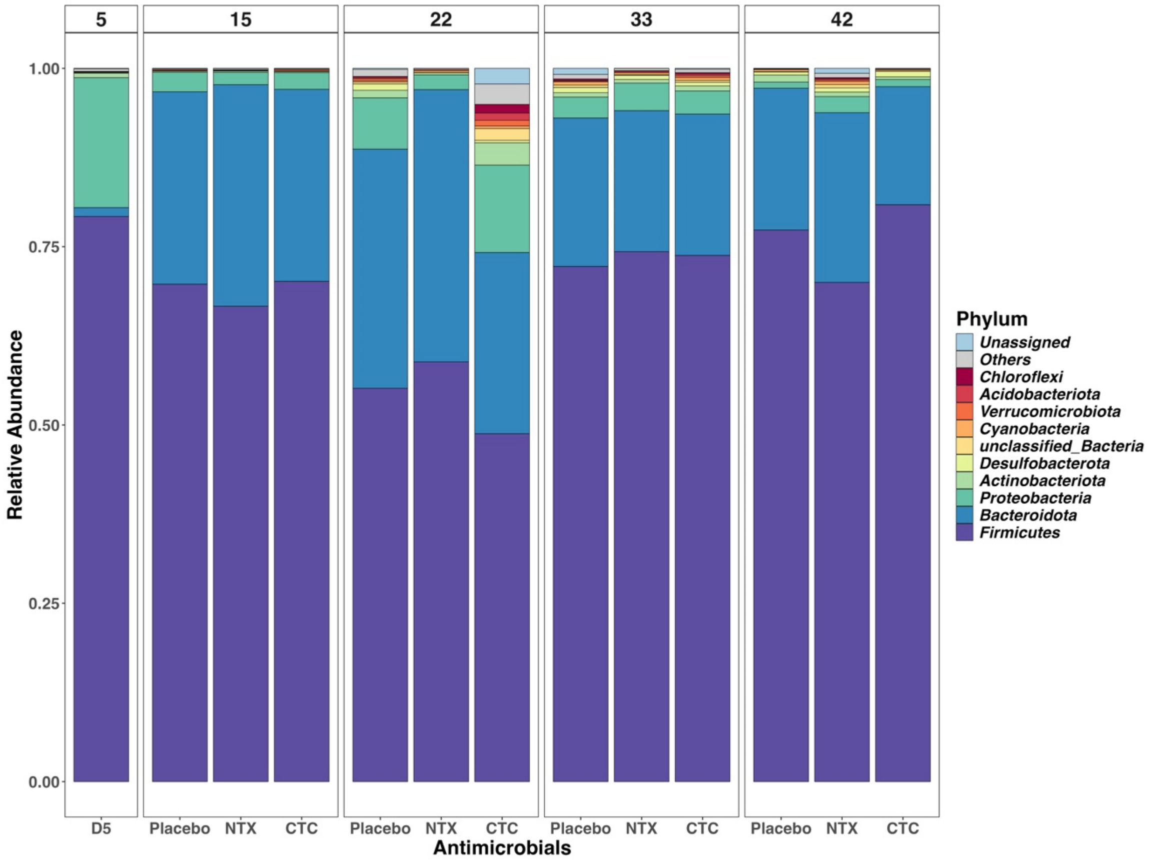

Supplementary Figure S6. Average relative abundances of the microbial community in the cecum at the phylum level. Birds were treated with either NTX (100 mg/kg) or CTC (50 mg/kg) continuously for 10 days (days 5 to 15) and were compared with a placebo group (fed a non-supplemented basal diet). 16S ribosomal RNA (rRNA) amplicon sequencing was performed on caecal contents collected on day 5 (*n* = 10 total), and on days 15, 22, 33, and 42 from six randomly picked broilers per group (*n* = 1 per replicate). Taxa with a relative abundance of < 1% were grouped into “Others”.

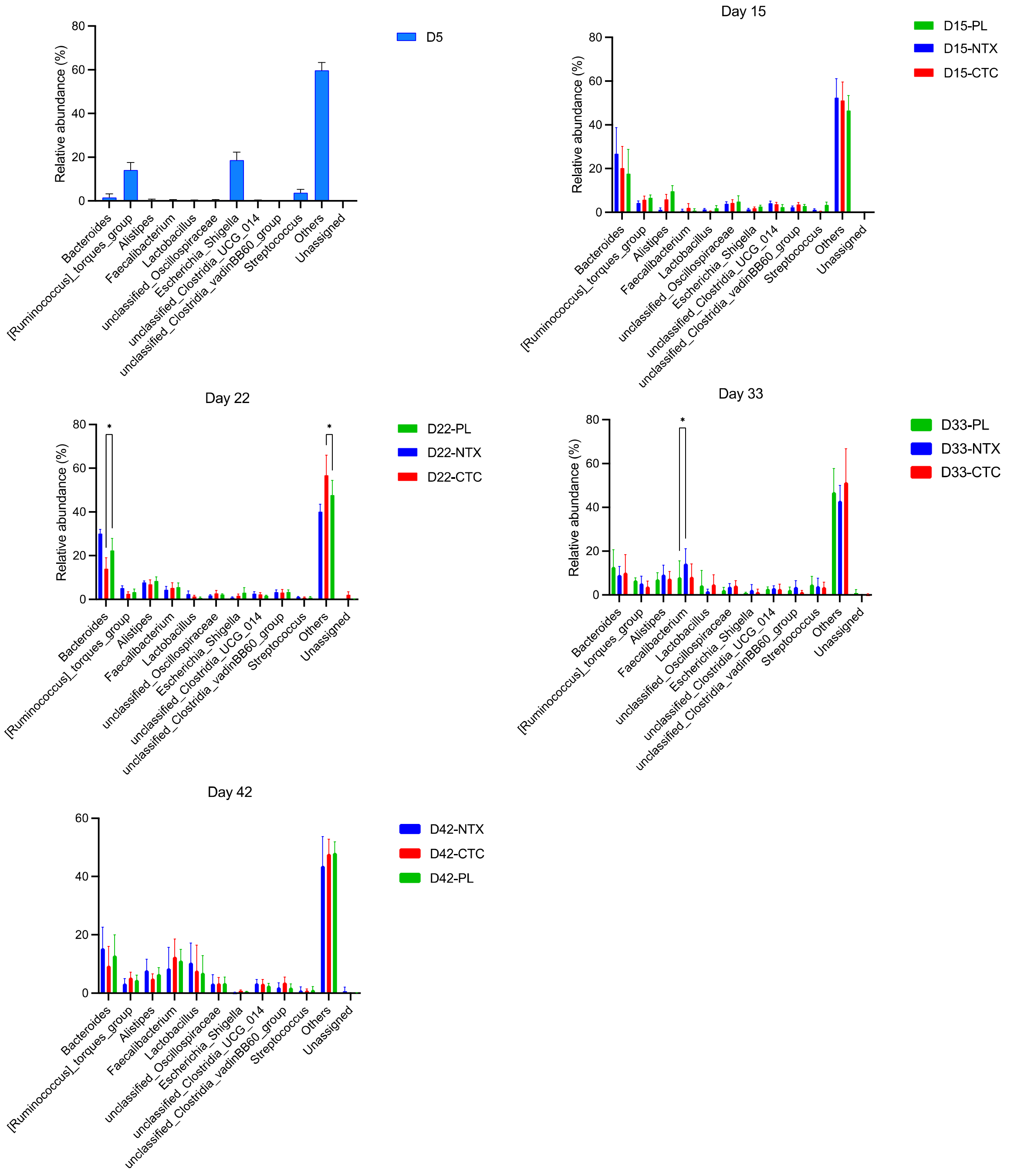

Supplementary Figure S7. Average relative abundances of the microbial community in the cecum at the genus level on different sampling days. Birds were treated with either NTX (100 mg/kg) or CTC (50 mg/kg) continuously for 10 days (days 5 to 15) and compared with a placebo (PL) group (fed a non-supplemented basal diet). 16S ribosomal RNA (rRNA) amplicon sequencing was performed on caecal contents collected on day 5 (n = 10 total), and on days 15, 22, 33, and 42 from six randomly picked broilers per group (n = 1 per replicate). Taxa with a relative abundance of < 1% were grouped into “Others”. Data are represented as mean ± SEM. Statistical differences were determined by two-way ANOVA with Dunnett’s multiple comparisons test using GraphPad Prism 10.0.3. Asterisks denote significant differences compared to the placebo group (*, $p < 0.05$).

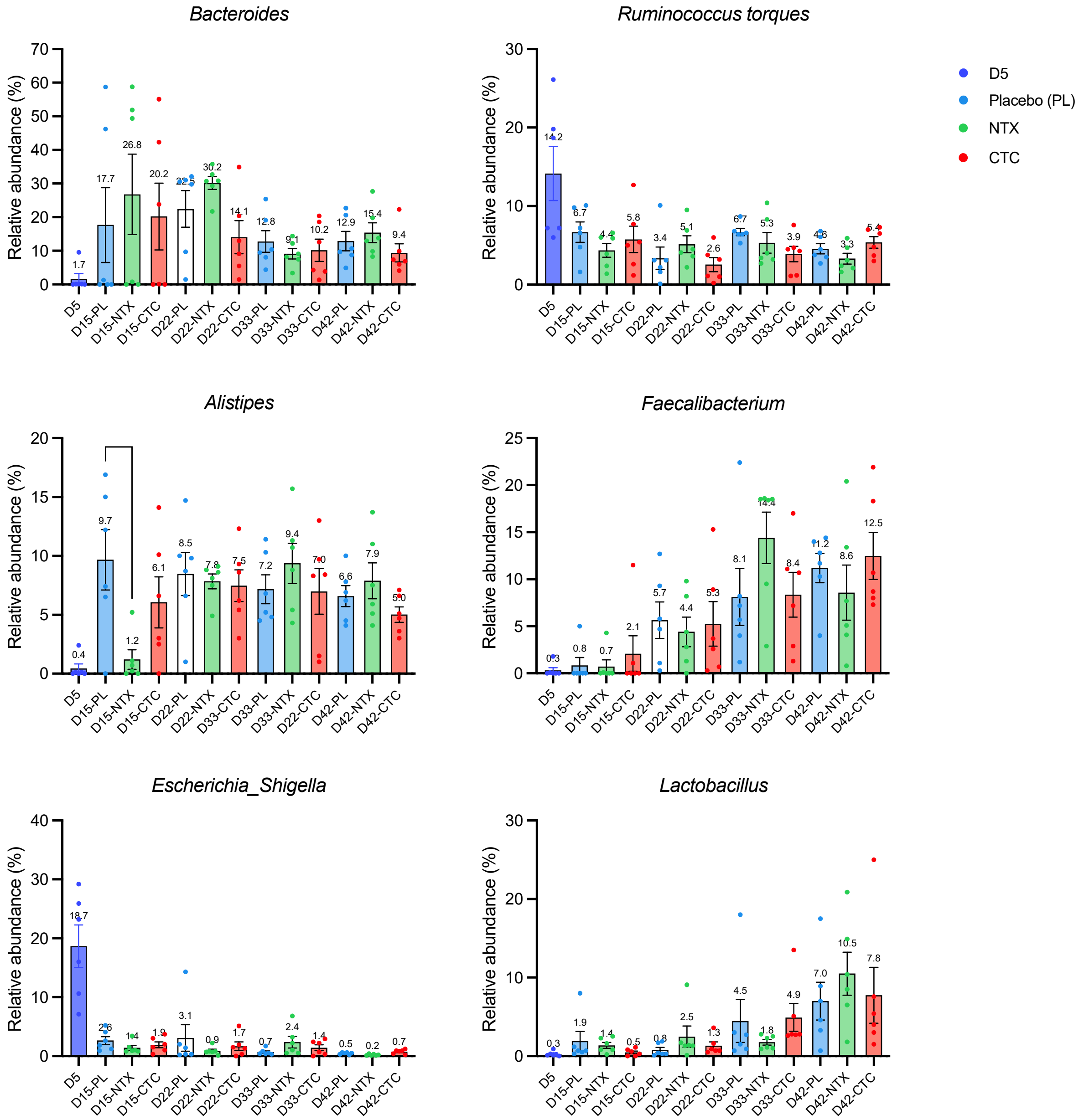

Supplementary Figure S8. Average relative abundances of the predominant genus in the microbial community in the cecum. Birds were treated with either NTX (100 mg/kg) or CTC (50 mg/kg) continuously for 10 days (days 5-15) and compared with a placebo (PL) group (fed a non-supplemented basal diet). 16S ribosomal RNA (rRNA) amplicon sequencing was performed on caecal contents collected on day 5 (n = 10 total), and on days 15, 22, 33, and 42 from 6 randomly picked broilers per group (n = 1 per replicate). Taxa with a relative abundance of < 1% were grouped into "Others". Data are represented as mean ± SEM. Statistical differences were determined by ordinary one-way ANOVA with Šídák's multiple comparisons test using GraphPad Prism 10.0.3. Asterisks denote significant differences compared to the placebo group on the same day (*, $p < 0.05$).

Supplementary Table S2. List of pathogenic bacterial strains and culture conditions used in this study.

Collection no.	Species	Gram strain	Isolated from	Growth conditions			
				Temp (°C)	Atmosphere	Broth	Agar
ATCC 25922	*Escherichia coli*	-	NA	37	Aerobic	CAMHB	MHA
DSM 103262	*Escherichia coli APEC O1:K1*	-	Chicken	37	Aerobic	CAMHB	MHA
DSM 103263	*Escherichia coli APEC O78:K80*	-	Chicken	37	Aerobic	CAMHB	MHA
DSM 4883	*Salmonella gallinarum*	-	Unknown	37	Aerobic	CAMHB	MHA
DSM 17420	*Salmonella enteritidis*	-	Unknown	37	Aerobic	CAMHB	MHA
DSM 19587	*Salmonella typhimurium*	-	Chicken	37	Aerobic	CAMHB	MHA
NCTC 5799	*Salmonella kentucky*	-	NA	37	Aerobic	CAMHB	MHA
DSM 23247	*Yersinia enterocolitica*	-	Unknown	37	Aerobic	CAMHB	MHA
DSM 30016	*Aeromonas hydrophila*	-	Surface water	25	Aerobic	CAMHB	MHA
DSM 13414	*Bordetella bronchiseptica*	-	Dog lung	37	Aerobic	CAMHB	MHA
DSM 30052	*Edwardsiella tarda*	-	Human faeces	25	Aerobic	CAMHB	MHA
DSM 16031	*Pasteurella multocida*	-	Pig	37	Aerobic	CAMHB+5% HB	MHA+5%SB
DSM 22433	*Actinobacillus suis*	-	Porcine	37	Aerobic	CAMHB+5% HB	Chocolate Agar
DSM 24114	*Campylobacter jejuni*	-	Human outbreak	37	Microaerophilic	CAMHB+5% HB	MHA+5%SB
DSM 11375	*Campylobacter lari*	-	Herring gulls	37	Microaerophilic	CAMHB+5%HB	Columbia agar + 5%SB
DSM 15997	*Ornithobacterium rhinotracheale*	-	Turkey	37	Microaerophilic	CAMHB+5% HB	Columbia Agar+5%SB
NCTC 8237	*Clostridium perfringens Type A*	+	NA	37	Anaerobic	Fastidious anaerobe broth	Brucella agar+5%SB
NCTC 10719	*Clostridium perfringens Type C*	+	NA	37	Anaerobic	Fastidious anaerobe broth	Brucella agar+5%SB

Collection no.	Species	Gram strain	Isolated from	Growth conditions			
				Temp (°C)	Atmosphere	Broth	Agar
DSM 1296	*Clostridioides difficile*	+	Unknown	37	Anaerobic	Fastidious anaerobe broth	Brucella agar+5%SB
DSM 346	*Staphylococcus aureus*	+	Unknown	37	Aerobic	CAMHB	MHA
DSM 20600	*Listeria monocytogenes*	+	Rabbit	37	Aerobic	CAMHB+5% HB	MHA+5%SB
DSM 5055	*Erysipelothrix rhusiopathiae*	+	Pig	37	Aerobic	CAMHB+5% HB	MHA+5%SB
DSM 20576	*Streptococcus iniae*	+	Dolphin abscess	25	Microaerophilic	CAMHB+5%HB	MHA+5%SB
DSM 9682	*Streptococcus suis*	+	Pig	37	Aerobic	CAMHB+5% HB	MHA+5%SB
DSM 15349	*Streptococcus gallinaceus*	+	Chicken sepsis	37	Aerobic	CAMHB+5% HB	MHA+5%SB

APEC, avian pathogenic *E. coli*. NA, not applicable. CAHMA, cation-adjusted Mueller-Hinton agar; MHA, Mueller-Hinton agar; SB, defibrinated sheep blood; HB, lysed horse blood.

Supplementary Table S3. NTX activity against wild-type drug-resistant *E. coli* isolates with specific resistance genes.

Wild-type strain	Species	Resistant gene	MIC of NTX (μg/mL)			
			**NTX**	**Colistin**	**Meropenem**	**Tigecycline**
NP 206	*E. coli*	*mcr*-1	4	4	<0.125	0.5
NP 301	*E. coli*	*mcr*-1	4	4	<0.125	0.5
NP 216	*E. coli*	*mcr*-1	4	8	<0.125	0.5
3R	*E. coli*	NDM-5	4	0.5	>64	0.5
8R	*E. coli*	NDM-5	4	0.5	>64	0.5
SPNP 706	*E. coli*	*tet*X4	8	4	<0.125	16
SPNP 724	*E. coli*	*tet*X4	8	8	<0.125	16
SPNP 731	*E. coli*	*tet*X4	8	4	<0.125	16

MIC, minimum inhibitory concentration; NTX, nitroxoline; *mcr*-1, confers resistance to colistin; NDM-5 is responsible for one of the main mechanisms of carbapenem resistance; *te*tX4 mediates resistance to tigecycline.

Supplementary Table S4. Antibacterial activity of NTX against reference *E. coli* and *Salmonella* spp. under 37 °C and 42 °C.

**Collection no.**	**Species**	**Gram strain**	**MIC of NTX (µg/mL)**	
			**37 °C**	**42 °C**
ATCC 25922	*Escherichia coli*	-	4, 2, 4	2, 2, 2
DSM 103262	*Escherichia coli APEC O1:K1*	-	2, 4, 4	2, 1, 2
DSM 103263	*Escherichia coli APEC O78:K80*	-	4, 4, 4	2, 2, 4
DSM 4883	*Salmonella enterica Gallinarum*	-	2, 1, 2	0.5, 0.5, 1
DSM 17420	*Salmonella enterica Enteritidis*	-	2, 2, 4	1, 0.5, 2
DSM 19587	*Salmonella enterica Typhimurium*	-	4, 4, 8	2, 1, 4
NCTC 5799	*Salmonella enterica Kentucky*	-	4, 4, 4	2, 1, 2

The minimum inhibitory concentration (MIC) values are distinguished by colour, indicating different atmospheric conditions: aerobic (black), microaerophilic (blue), and anaerobic (red).

Supplementary Table S5. The wild-type isolates screened for NTX-resistant mutants.

Strain	Species	Resistance phenotype	Frequency of resistance at 2 x MIC
ASS3	*S. enterica*	Ciprofloxacin, Tigecycline	1.9×10^{-7}
LCS2	*S. enterica*	Ciprofloxacin, Tigecycline	No mutant found
MCS11	*S. enterica*	Ampicillin, Gentamicin, Ciprofloxacin, Tigecycline	6.7×10^{-7}
MCS12	*S. enterica*	Ampicillin, Gentamicin, Ciprofloxacin, Tigecycline	2.2×10^{-7}
SCS10	*S. enterica*	Ampicillin, Tigecycline	No mutant found
APNP126	*E. coli*	Ampicillin, Gentamicine, Ciprofloxacin, Colistin, Tigecycline	2.0×10^{-7}
APNP426	*E. coli*	Ampicillin, Gentamicin, Ciprofloxacin, Colistin	No mutant found
ASNP649	*E. coli*	Ampicillin, Gentamicin, Ciprofloxacin, Tigecycline	No mutant found
SSNP567	*E. coli*	Ampicillin, Cefotaxime, Ceftazadime, Gentamicin, Ciprofloxacin	6.9×10^{-6}
SSNP585	*E. coli*	Ampicillin, Ciprofloxacin	No mutant found
LSNP284[a]	*E. coli*	Ampicillin, Tigecycline	NA

a, The *E. coli* LSNP284 strain was only selected for multi-step resistance study by serial passage assay. NA, not applicable. MIC, minimum inhibitory concentration. No mutations were observed at the concentrations of 4× or 8× MIC (16 and 32 μg/mL, respectively).

Supplementary Table S6. MICs of NTX for parental strains and mutants on day 0 and day 14.

Strain	Parent/Mutant	MIC of NTX (μg/mL)	
		Day 0	Day 14
E. coli ATCC 25922	Parent	4	4
Mut ATCC-D13	Mutant	16	8
Mut ATCC-D14	Mutant	16	4-8
E. coli DSM 103263	Parent	4	4
Mut DSM-D8	Mutant	32	8
Mut DSM-D18	Mutant	32	16

Four colonies from each strain were selected for minimum inhibitory concentration (MIC) determination using the microbroth dilution method.

Supplementary Table S7. Hematologic parameters in broiler chickens after fed with 50 ppm and 500 ppm NTX for 7 consecutive days.

Haematological Parameter	Units	Placebo (Basal diet)	NTX50 (50 mg/kg NTX diet)	NTX500 (500 mg/kg NTX diet)
RBC	10^{12} cells/L	2.22 ± 0.05	2.14 ± 0.12	2.19 ± 0.07
HGB	g/L	77.67 ± 4.04	71.00 ± 9.85	74.67 ± 6.11
HCT	%	31.97 ± 1.29	29.97 ± 2.91	31.00 ± 1.77
MCV	fL	131.27 ± 2.47	131.23 ± 2.28	132.2 ± 2.33
MCHC	g/L	292.67 ± 22.05	257.67 ± 50.77	276.33 ± 30.89
WBC	10^9 cells/L	45.69 ± 2.14	44.58 ± 2.05	44.40 ± 1.66
Neu	%	36.47 ± 2.29	35.7 ± 4.17	32.2 ± 1.01
Lym	%	50.33 ± 3.78	50.47 ± 3.99	54.47 ± 2.20
Mon	%	10.5 ± 1.47	10 ± 0.46	10.27 ± 1.30
Eos	%	1.97 ± 0.99	3.33 ± 0.15	2.17 ± 0.25
Bas	%	0.73 ± 0.30	0.5 ± 0.17	0.9 ± 0.52
PLT	10^9 cells/L	2.67 ± 2.08	4 ± 1.73	3.33 ± 0.58
MPV	fL	7.73 ± 0.76	8.53 ± 0.93	8.6 ± 0.36

RBC, Red Blood Cells; HGB, Haemoglobin; HCT, Haematocrit; MCV, Mean Corpuscular Volume; MCHC, Mean Corpuscular Haemoglobin Concentration; WBC, White Blood Cells; Neu, Neutrophils; Lym, Lymphocytes; Mon, Monocytes; Eos, Eosinophils; Bas, Basophils; PLT, Platelets; MPV, Mean Platelet Volume. The data are expressed as mean ± SD (n = 3). Statistical differences were determined by ordinary one-way ANOVA with Dunnett multiple comparisons test using GraphPad Prism 10.0.3. No significant difference was found.

Supplementary Table S8. NTX concentrations in plasma of adult chickens following a single oral administration at 100 mg/kg BW (n = 7).

Time (h)	Plasma concentration (ng/mL)							Mean	SD
	No. 1	No. 2	No. 3	No. 4	No. 5	No. 6	No. 7		
0.083	3285	11120	4225	4780	20535	5780	15290	9287.86	6585.00
0.25	45311	19990	15455	12510	28410	8340	24320	22048.00	12338.22
0.5	17695	23890	22150	12820	26650	16160	27225	20941.43	5501.28
1	18820	33445	22375	14225	26825	11210	29915	22402.14	8194.63
2	13380	23485	43495	20305	27520	24525	18495	24457.86	9559.13
3	6315	11825	26880	15230	16010	24920	9900	15868.57	7603.21
4	3675	7915	8870	14410	5980	7860	4960	7667.14	3491.03
5	4485	3855	3820	9135	5305	6975	5090	5523.57	1921.36
6	4925	2175	3630	5460	3290	3025	2125	3518.57	1278.19
8	7685	2280	1465	1530	4010	2475	1750	3027.86	2230.01
12	1797.1	240.5	175.5	275.2	685.6	676	113.8	566.24	590.13
24	1429.3	73.6	24.9	34.8	23.7	63.3	17.6	238.17	525.67
36	279.2	22.6	33.4	12.4	11.5	23.2	6.2	55.50	99.06
48	203.8	14.7	20.2	BLOQ	9	8.3	BLOQ	51.20	85.44

BLOQ: below the limit of quantification (5 ng/mL).

Supplementary Table S9. NTX concentrations in plasma and tissues of young chicks following a single oral administration at 30 mg/kg BW.

Time (h)	NTX concentration (ng/mL)			
	Plasma	Liver	Kidney	Muscle
0.083	5002.67 ± 3329	438.43 ± 447.04	615.33 ± 531.56	413.73 ± 259.72
0.25	8458.33 ± 1946.97	1131.66 ± 651.72	1035.98 ± 485.36	905.5 ± 434.49
0.5	7087.5 ± 5339.38	2395.02 ± 1743.28	480.72 ± 255.39	511.43 ± 374.31
1	2777.33 ± 262.67	2588.02 ± 1815.09	833.92 ± 636.17	210.11 ± 126.11
2	1618.38 ± 788.08	4004.27 ± 4019.56	598.15 ± 132.66	136.91 ± 56.44
3	626.98 ± 395.06	2133.71 ± 790.58	351.79 ± 151.2	226.01 ± 188.67
4	809.45 ± 277.02	2749.42 ± 1355.99	151.68 ± 48.75	91.65 ± 132.1
5	481.12 ± 314.81	3391.75 ± 1767.76	198.73 ± 155.35	65.44 ± 22.99
8	47.75 ± 15.25	178.88 ± 144.79	38.56 ± 25.45	11.15 ± 1.97
12	30.5 ± 16.73	119.32 ± 132.15	72.601 ± 41.9	37.48 ± 15.59
24	15.27 ± 9.08	BLOQ	28.81 ± 18.23	BLOQ

BLOQ: below the limit of quantification (10 ng/g). Values are presented as mean ± SD (*n* = 6).

Supplementary Table S10. NTX residue (ng/g) in chicken tissues following withdrawal of feed containing 100 mg/kg NTX for 10 consecutive days (*n* = 6).

Withdrawal period (day)	Animal No.	Liver	Kidney	Muscle	Skin-plus-fat
0.25	1	45.86	17.10	25.14	191.78
	2	BLOQ	BLOQ	27.28	138.78
	3	BLOQ	BLOQ	23.12	151.11
	4	BLOQ	BLOQ	24.82	183.62
	5	BLOQ	BLOQ	14.68	171.81
	6	BLOQ	BLOQ	16.74	231.07
	Mean ± SD	NA	NA	21.96 ± 5.06	178.03 ± 32.67
1	1	BLOQ	BLOQ	BLOQ	51.89
	2	BLOQ	BLOQ	BLOQ	66.71
	3	BLOQ	BLOQ	BLOQ	121.33
	4	BLOQ	BLOQ	BLOQ	107.48
	5	BLOQ	BLOQ	BLOQ	94.71
	6	BLOQ	BLOQ	BLOQ	103.46
	Mean ± SD	NA	NA	NA	90.93 ± 26.38
3	1	BLOQ	BLOQ	BLOQ	37.83
	2	BLOQ	BLOQ	BLOQ	40.76
	3	BLOQ	BLOQ	BLOQ	41.97
	4	BLOQ	BLOQ	BLOQ	35.63
	5	BLOQ	BLOQ	BLOQ	62.65
	6	BLOQ	BLOQ	BLOQ	50.12
	Mean ± SD	NA	NA	NA	44.83 ± 10.04
5	1	BLOQ	BLOQ	BLOQ	28.92
	2	BLOQ	BLOQ	BLOQ	38.36

Withdrawal period (day)	Animal No.	Liver	Kidney	Muscle	Skin-plus-fat
	3	BLOQ	BLOQ	BLOQ	28.16
	4	BLOQ	BLOQ	BLOQ	35.01
	5	BLOQ	BLOQ	BLOQ	55.39
	6	BLOQ	BLOQ	BLOQ	95.47
	Mean ± SD	NA	NA	NA	46.88 ± 25.77
8	1	BLOQ	BLOQ	BLOQ	20.07
	2	BLOQ	BLOQ	BLOQ	18.29
	3	BLOQ	BLOQ	BLOQ	6.31
	4	BLOQ	BLOQ	BLOQ	26.28
	5	BLOQ	BLOQ	BLOQ	26.19
	6	BLOQ	BLOQ	BLOQ	34.17
	Mean ± SD	NA	NA	NA	21.88 ± 9.46

BLOQ, below the limit of quantification (10 ng/g). NA, not applicable.
